# Big Data Analytics Framework for Early Breast Cancer Detection Using Optimized MapReduce and Deep Learning on Large‐Scale Mammographic Data

**DOI:** 10.1155/ijbi/8325888

**Published:** 2026-09-30

**Authors:** Mohammed M. Ahmed, Ehab Hassanien

**Affiliations:** ^1^ Faculty of Computing and Information Technology Sohar University Sohar City Oman soharuni.edu.om; ^2^ Faculty of Computers and Artificial Intelligence University of Sadat City Sadat City Egypt usc.edu.eg; ^3^ Faculty of Computers and Artificial Intelligence Cairo University Giza City Egypt cu.edu.eg

**Keywords:** BIRCH, breast cancer detection, deep learning classification, large-scale data, MapReduce

## Abstract

The paper presents a framework that is aimed at addressing the large‐scale data challenge with a particular focus on early prediction of breast cancer based on mammography image data. The abundance of large‐scale medical imaging data in healthcare has opened the way to intelligent diagnostic systems through its ability to analyze and process large‐scale mammography data. The proposed framework, the metaheuristic optimization based large‐scale breast cancer detection (MO‐LSBCD) framework provides a complete solution to the extraction of discriminative features that can be used to predict the disease correctly. Early and accurate diagnosis of breast cancer is one of the most critical factors in improving survival rates and reducing treatment costs for patients. The MO‐LSBCD framework is divided into three phases: (1) The CBIS‐DDSM mammography dataset consists of 10,239 image files with a size of 6 GB, but for classification, the analytical unit is the annotated region of interest (ROI), with 3568 ROI‐level abnormality records from 1566 unique patients (normal, malignant, benign, and benign‐without‐callback); (2) solving the large‐scale data problem by implementing an optimized MapReduce model to efficiently distribute and process the large volumes of breast cancer mammography images in parallel; and (3) effective deep learning techniques to classify breast cancer pathology. The experimental findings indicate that the enhanced MapReduce model reduces total processing time and improves F‐measure in comparison with the standard MapReduce model. Among different deep learning models tested on the CBIS‐DDSM dataset, the classification of EfficientNet‐B3 and ResNet‐50 was better with an accuracy of 99.64% and 98.91%, respectively, compared with the other models on all evaluation metrics. The results of this paper confirm that MO‐LSBCD framework can be used to provide a robust and scalable solution to large‐scale breast cancer detection to help clinicians in the early diagnosis of breast cancer and eventually help in the improved patient outcome.

## 1. Introduction

The high rate of development of new medical imaging technologies has resulted in an unprecedented increase in the amount of data generated by diagnostic devices, hospital information systems, and intelligent healthcare platforms. Massive analytics of medical data has become a pillar of contemporary intelligent healthcare systems that are capable of real‐time monitoring, predictive modeling, and automated decision‐making in a wide range of clinical settings. Specifically, big data analytics is of vital importance in deriving meaningful knowledge out of large and heterogeneous datasets of medical imaging.

Although several studies have employed MapReduce or deep learning for medical image analysis, most of them address scalability and classification independently and do not optimize the clustering process during distributed processing. The proposed MO‐LSBCD framework is therefore novel because it integrates optimized MapReduce with hybrid clustering strategies, namely slime mould algorithm combined Balanced Iterative Reducing and Clustering using Hierarchies clustering (BIRCH‐SMA) and Marine Predators Algorithm combined with slime mould algorithm (MPA‐SMA), and deep learning in a unified architecture for large‐scale mammographic data analysis.

In the clinical screening situation, digital mammography systems store huge amounts of breast images in extensive health care institutions. But processing these mammography images in real‐time is a significant computational challenge due to the sheer number of images involved in thousands of high‐resolution mammography scans with huge storage requirements. MO‐LSBCD can be used to solve this computational problem by using the combination of MapReduce and clustering technique of BIRCH‐SMA. Under the clustering method, the data partitioning of the pictures is carried out in the clustering process. One of the industries that have been greatly reshaped by the power of big data technologies is the field of healthcare, and oncology, in particular.

Breast cancer is one of the most prevalent and life‐threatening cancers among women worldwide, and its high prevalence has contributed to an increasing demand for highly accurate diagnostic methods and scalable automated screening systems. The increase in the 5‐year survival rate to more than 90% with an early‐stage diagnosis and a critically underaddressed late‐stage diagnosis is estimated. These issues prompt the adoption of a shift to data‐driven smart diagnostic systems, in which large‐scale analytics can be used to enhance accuracy, clinical performance, and operational effectiveness. The use of digital mammography and large‐scale databases of medical imaging makes possible the gathering of huge volumes of annotated data in hospitals, research centers, and public databases of medical imaging. These systems produce high‐resolution images and rich clinical metadata of tissue morphology, abnormality type, breast density, radiologist assessment, and confirmed pathology. Through large‐scale data, clinical stakeholders can optimize disease prediction pipelines, minimize false‐positive rates, and generally improve resource allocation in screening programs [1, 2].

Although such great and complicated medical imaging datasets have been developed, it is still hard to effectively use such large and complex medical imaging datasets. The data in healthcare imaging are heterogeneous in nature and originate with a variety of acquisition devices that run under different formats and communication protocols, making data integration and interoperability difficult [3, 4]. Moreover, medical image data are too large and dimensional to be efficiently processed by a single computer with limited processing capabilities [5, 6]. Besides, data privacy and data security must be ensured because patient records are sensitive information and cloud‐based platforms and distributed healthcare infrastructures are rapidly becoming more dependent [7, 8].

Paradigms of distributed processing like MapReduce have been extensively used to overcome big data analytics scalability challenges. Some of the research has tried to enhance the performance of MapReduce or recreating sequential analysis algorithms in distributed systems [9, 10]. Nonetheless, many of the current MapReduce‐based systems are based on naive implementation of traditional algorithms, not inherently designed to be used in iterative optimization processes, and especially in clustering and intelligent classification problems. As a result, their performance, convergence properties, and scalability can be compromised when using large‐scale medical imaging data. The conventional disease classification of breast cancer generally classifies lesions into benign and malignant lesions further classified based on type of abnormality, that is, calcifications and masses. Even though the recent state‐of‐the‐art deep learning models have shown promising results in automated breast cancer detection, their integration into optimized large‐scale distributed analytics platforms has been a limited area of discussion in literature.

The literature on most of the existing works either focuses on intelligent disease classification or distributed processing separately, and not on a unified architecture that integrates scalable computation with hybrid optimization strategies. Thus, there is a definite gap in research to create unified large‐scale data that can simultaneously achieve distributed scalability, optimized clustering, and deep learning–based breast cancer detection in large‐scale medical imaging settings. To fill this gap, this paper will present a new metaheuristic optimization based large‐scale breast cancer detection (MO‐LSBCD) framework, which will improve the efficiency of distributed processing and will increase the accuracy of breast cancer prediction. The proposed framework maximizes the MapReduce paradigm by incorporating hybrid metaheuristic clustering mechanisms in both the mapper and reducer stages to effectively handle the mammography data of a large population and apply intelligent disease classification.

Unlike previous studies that use MapReduce or deep learning independently, this work proposes a unified MO‐LSBCD framework for large‐scale mammographic data analysis. The novelty of this study lies in integrating BIRCH‐SMA into the mapper stage and MPA‐SMA into the reducer stage to improve clustering and data partitioning during MapReduce processing, thereby enhancing both computational efficiency and breast cancer classification performance.

The contributions of this paper are as follows:

a. A new proposed MO‐LSBCD framework is introduced for large‐scale mammographic breast cancer detection using an optimization‐driven MapReduce design.
b. The BIRCH‐SMA mapper optimization strategy is developed to partition efficiently and optimize their cluster centroids through slime mould algorithm (SMA).
c. The MPA‐SMA reducer optimization strategy is developed to explore the global clustering space using MPA and refine the final solution using SMA.
d. A comparative evaluation is presented for different algorithmic configurations and deep learning–based classification models.

The structure of the paper is outlined as follows: Section 2 delves into related works, whereas Section 3 covers preliminaries of fundamental concepts. Section 4 presents the proposed framework, MO‐LSBCD, designed to address large‐scale data challenges in predicting breast cancer diseases. Performance evaluations are detailed in Section 5, and the results from the experiments are examined in Section 6. The paper concludes in Section 7, where it proposes areas for future investigation.

## 2. Literature Review

In this section, the authors briefly describe the recent studies and advances in large‐scale data to smart healthcare systems based on medical imaging. Agrawal et al. [11] present an enhancement‐aware mammography pipeline showing that preclassification optimization can substantially improve downstream learning efficiency. The CbcErDL framework integrates two‐stage CLAHE enhancement, DCT‐based image reduction, and deep learning classification, demonstrating that dimensionality can be reduced while preserving diagnostically informative patterns. This is particularly relevant for large‐scale screening settings because representation compression reduces computational load without discarding clinically meaningful structures.

Another element proposed by the authors was an architectural framework of smart healthcare systems and various innovative technology challenges were discussed. Additional resource applications, clinical data sources and analytic capabilities were discussed further in [12]. Jabeen et al. [13] advance Curated Breast Imaging Subset of Digital Database for Screening Mammography (CBIS‐DDSM)–oriented classification by combining enhanced deep features with Equilibrium‐Jaya controlled Regula‐Falsi feature selection (FS). The method emphasizes selective retention of discriminative attributes and suppression of redundant features, thereby improving robustness and efficiency. This study highlights that model performance in mammography depends not only on backbone architecture but also on feature curation and optimization strategy.

Interoperability is one of the main challenges that have been stated in big‐scale analytics of medical data. The heterogeneity of imaging devices, communication protocols, and data formats presents a challenge in integrating and harmonizing data between varied clinical sources. Data interoperability can be ensured through standardization, common data models, and well‐defined communication protocols. Wang [14] provide a recent review oncology that synthesizes modern deep learning–assisted x‐ray mammography and identifies key research priorities including generalizability, interpretability, privacy‐aware deployment, and clinically reliable early prediction workflows. Incorporating this survey establishes a current benchmark context for our study and positions MO‐LSBCD against contemporary evidence rather than only legacy big‐data analytics references.

These interoperability concerns are even more acute in large‐scale hospital networks applications, where Al‐Maashri et al. [15] highlighted issues related to data nonhomogeneity and the necessity to deploy high‐level analytics infrastructure. One of the limitations is that it can be complicated to integrate various clinical data sources and ensure data quality, which may require effective data preprocessing methods and standardized data formats.

Scalability, in addition to interoperability, becomes another important issue when it comes to dealing with large‐scale medical imaging data analytics. The growing amount of images systems and the growing volume of mammography data need scalable analytics systems and distributed computing systems. Nevertheless, the scalability of data analytics systems can be obstructed by the limitations of network bandwidth, processing power, as well as storage capacity. A survey on big data analytics in healthcare systems, with a focus on the challenges and potential solutions of scalable analytics, is provided by Yu et al. [16]. The problem of scalability is further complicated by the real‐time processing demands. Many clinical screening applications rely on real‐time analytics, which allows timely and acted upon decisions and actions. A survey of the healthcare data analytics field by Abbasi et al. [17] revealed such limitations as the necessity to use efficient stream processing methods, low‐latency analytics systems, and optimized sources allocation. Furthermore, reliability and stability of real‐time analytics systems should be discussed to guarantee the accuracy and timeliness of diagnostic insights.

To overcome these scalability and real‐time problems, the selection of appropriate data analytics techniques should be meticulously chosen. A general survey of data analytics in medical imaging systems, including data management, data mining, and data visualization methods was presented by Mishra et al. [18]. One limitation is that it is difficult to select the appropriate data analytics algorithms for different types of medical imaging data and areas of clinical application, without an in‐depth understanding of the data characteristics and specific requirements. To complement the choice of analytics methods, efficient handling of data is similarly significant to large‐scale medical data analytics. Mohammed et al. [19] have reviewed big data analytics regarding healthcare systems, with limitations including the complexity of data preprocessing, the complexity of data integration, and the need to have scalable data retrieval and data storage systems. Also, cost and scalability of storage infrastructure and data retention policies should be taken into consideration to ensure maximum utilization of resources and cost‐effectiveness.

Abulfaraj [20] introduce a cross‐center vision‐language transformer for robust mammography diagnosis, directly addressing domain shift across institutions. This is a critical addition because many high‐performing single‐center models degrade under cross‐site data heterogeneity. Including this study strengthens the motivation for scalable frameworks such as MO‐LSBCD that should be evaluated under both performance and deployment robustness constraints.

Downsides are that there is a risk of data breaches, unauthorized access, and even a violation of healthcare privacy laws like HIPAA. To reduce these risks, it is necessary to implement strong security measures, including encryption of data, control of access, and the use of anonymization techniques. In addition to security, the credibility of analytics systems is based on their interpretability and explainability. There are difficulties in applying complex machine learning algorithms to medical imaging data because they are black boxes. To overcome these limitations, Puthal et al. [21] proposed a deep learning–based methodology for healthcare‐enabled smart diagnostic systems with the importance of explainable AI and model interpretability. Explainable AI methods, including rule‐based models and transparent algorithms can be used to achieve a better understanding of the decision‐making process and increase trust in the diagnostic analytics results.

All these obstacles are based on underlying computational and infrastructure constraints. The large‐scale medical imaging system and big data analytics system can consume many computational resources, such as high‐performance computing systems and distributed processing frameworks. Moreover, network connectivity and cloud infrastructure availability and reliability have a significant impact on facilitating efficient clinical data processing. In the light of the cost, scalability, and availability of computational resources, it is required to ensure the feasibility and efficiency of large‐scale medical imaging data analytics systems that are designed to detect and classify breast cancer.

Previous studies mainly focus on either distributed data processing or deep learning–based classification, but they do not combine these tasks in a unified optimization framework. In contrast, the proposed MO‐LSBCD framework jointly addresses scalability, clustering optimization, and classification, which makes it different from existing approaches and improves the overall performance for large‐scale mammographic analysis.

In recent years, the analysis of mammograms has been studied, and deep learning–based techniques have proven to be effective in the classification of images and the prediction of diagnosis. For instance, Agarwal et al. and Wang highlighted the importance of deep learning in breast cancer detection from mammograms, and Perea‐Jacobo et al. [22], Michael et al. [23], and López‐Cabrera et al. [24] investigated classification accuracy on mammograms and various deep learning models. Although these methods have demonstrated good accuracy in medical image analysis, they are typically developed as independent classification models and is primarily aimed at enhancing diagnostic accuracy, but not the computational burden of very large‐scale mammographic data. The proposed MO‐LSBCD framework, on the other hand, does not require a specific classification model. It is intended to be an integrated large‐scale analytical framework, which integrates distributed data processing, optimized clustering and metaheuristic refinement, and classification. MO‐LSBCD is different from these approaches in three ways. First, it highlights scalability, as it includes a large‐scale data processing strategy that allows for the analysis of high‐volume mammographic data in a more efficient manner. Second, it proposes clustering optimization using BIRCH‐SMA and MPA‐SMA, which enhances the segmentation quality and parameter tuning over the conventional clustering or static classification pipeline. Third, it combines these components into a single workflow with the goal of enhancing the classification accuracy while keeping the computational cost low. Thus, the existing works focus on enhancing the classification accuracy of mammograms, whereas the proposed MO‐LSBCD framework offers a comprehensive and scalable approach to large‐scale mammographic data analysis.

In summary, the reviewed literature reveals several interconnected challenges: interoperability, scalability, data management, security, and computational limitations. Although existing studies have addressed these issues individually, there remains a gap in providing an integrated solution that optimizes large‐scale data processing while addressing domain‐specific applications. Therefore, this paper proposes a new framework for breast cancer detection based on large‐scale data (MO‐LSBCD) that addresses two main problems: (1) solving issues of MapReduce through optimization, and (2) enabling early detection of breast cancer diseases. Table 1 summarizes the related work and their limitations.

**Table 1:** Related works and their research gaps.

**Reference**	**Contributions**	**Research gaps**
Agrawal et al. [11]	Proposed CbcErDL pipeline combining CLAHE enhancement, DCT‐based reduction, and DL classification to improve mammogram CAD efficiency.	Focuses on pipeline‐level gains but provides limited evidence on cross‐center generalization and deployment robustness.
Wang [14]	Delivered a recent DL‐assisted mammography review summarizing modern methods and key priorities (generalizability, interpretability, clinical reliability).	As a survey, it does not provide a single new deployable model or unified experimental benchmark implementation.
Al‐Maashri et al. [15]	Reviewed big data analytics in smart cities.	Integration and harmonization of diverse data sources, and scalability of real‐time processing.
Yu et al. [16]	Presented a survey on big data analytics for systems, emphasizing challenges and solutions for scalable analytics.	Scalability, distributed computing, and cloud‐based analytics platforms.
Abbasi et al. [17]	Data analytics, identifying challenges such as efficient stream processing, low‐latency analytics, and optimized resource allocation.	Scalability of stream processing, real‐time analytics, and resource optimization.
Mishra et al. [18]	Presented a comprehensive of data analytics for the Internet of Things.	Data quality, integration, and selection of appropriate analytics algorithms.
Mohammed et al. [19]	Big data analytics for systems.	Complexity of data preprocessing, integration challenges, and scalability of storage.
Abulfaraj [20]	Proposed a cross‐center vision‐language transformer to improve robustness of mammography diagnosis.	Advanced architecture may require higher compute resources and broader external validation for routine clinical deployment.
Puthal et al. [21]	Proposed a deep learning–based approach for enabled smart cities, emphasizing the importance of explainable AI and model interpretability.	Interpretability and explainability of machine learning algorithms, availability of labeled training data.
Agarwal et al. [25]	Reviews and discusses deep learning approaches for mammography‐based breast cancer detection.	Focuses mainly on detection rather than integrated scalable analytics.
Perea‐Jacobo et al. [22]	Demonstrates deep learning–based classification performance on mammography images.	Does not integrate optimization or distributed analytics.
Michael et al. [23]	Shows performance of deep learning under small‐data conditions.	Generalization and scalability remain limited.
López‐Cabrera et al. [24]	Shows the applicability of deep learning for classification from mammography.	Does not address large‐scale processing or hybrid optimization.

## 3. Preliminaries

In this section, we will define a few terms before we introduce our method. This section includes the backgrounds of different techniques that used in proposed framework clustering technique Balanced Iterative Reducing and Clustering using Hierarchies (BIRCH) (Section 3.1), optimization of SMA (Section 3.2), Marine Predators Algorithm (MPA) (Section 3.3), definition of large‐scale data (Section 3.4), the MapReduce model (Section 3.5). Finally, Section 3.6 provides a definition of FS.

### 3.1. BIRCH

BIRCH [26] is a hierarchical clustering method designed to handle large datasets efficiently. It constructs a compact summary of the data using a Clustering Feature (CF) tree, which allows the algorithm to avoid repeated full scans of all data points. This property makes BIRCH especially suitable for large‐scale datasets where memory efficiency and computational scalability are critical. Instead of representing every sample independently, BIRCH compresses local information into CF vectors that preserve cluster summaries. The method is particularly useful in applications where data arrive in large quantities and require fast clustering with limited computational overhead. In the context of large‐scale mammographic analysis, BIRCH can be used to summarize local data partitions before further optimization. This enables the framework to reduce processing burden while retaining the essential structure of the data distribution. As a result, BIRCH provides a practical foundation for distributed or partition‐based clustering in large‐scale medical image analytics.

### 3.2. SMA

The SMA [27] is a bioinspired metaheuristic optimization method inspired by the foraging behavior of slime molds. It models how slime molds search for food sources and move through the environment, enabling a balance between exploration and exploitation during optimization. SMA is particularly effective for nonconvex and multimodal optimization problems where classical methods may become trapped in local optima. The algorithm updates candidate solutions according to adaptive movement rules and dynamic search behavior, helping the search process escape poor regions and refine promising ones. In this framework, SMA is used to optimize local cluster representatives obtained from BIRCH, improving the quality of intermediate cluster centers before global aggregation. This makes SMA highly suitable for large‐scale mammographic clustering where local cluster quality strongly affects overall performance. The algorithm has been demonstrated to be competitive in parameter optimization and structural search tasks because of its robust search dynamics and low dependence on problem‐specific assumptions.

### 3.3. MPA

The MPA [28] is a metaheuristic optimization method inspired by predator–prey dynamics in marine ecosystems. It simulates the coordinated movement of predators and prey to explore the search space and identify high‐quality candidate solutions. MPA is particularly useful for complex optimization problems with nonlinear landscapes, multiple local optima, and large search dimensions. Unlike many classical search methods, MPA balances exploration and exploitation through biologically motivated interaction strategies, allowing it to identify diverse promising regions before refining them. In the proposed MO‐LSBCD framework, MPA is used at the reducer stage to optimize the global clustering configuration generated by Mapper outputs. This enables the method to integrate local cluster summaries into a globally optimized representation of the mammographic dataset. As a result, MPA contributes to more robust global optimization and improves the final clustering or grouping quality across large‐scale data partitions.

### 3.4. Large‐Scale Data

The term “Large‐Scale Data” [29] is used to describe the ever‐expanding, massive, and varied collections of data that are being generated today. It includes not only how much data exists, but also how quickly it is generated and collected, and how many different types of data are included in the analysis. Data mining is a common source of big data, and it can come in a variety of forms.

The analysis of large amounts of data has become crucial to modern industries. Online purchases, emails, scientific data, images, videos, health records, audio files, posts, search queries, social media interactions, and app‐generated data [30, 31]. The amounts of information that are captured, formed, stored, managed, shared, analyzed, and visualized in databases continue to grow exponentially, making it more challenging to do so using the tools that are typically associated with such systems.

The most crucial concerns for big data have been discussed.

#### 3.4.1. Major Concerns

Figure 1 shows the three main components of big data: variety, velocity, and volume [32], all of which call for a revolutionary leap forward from conventional approaches to data analysis [33]. Variety is the spice of big data. Structured, semistructured, and unstructured data are the three main categories that make up big data. Unstructured data is haphazard and difficult to analyze, whereas structured data can be directly imported into a data warehouse with appropriate tags already applied. Semistructured data does not have predetermined fields, but it does use tags to organize its constituent parts.

**Figure 1:**
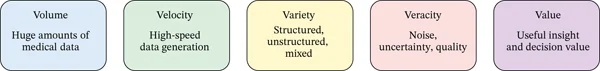
Large‐scale data components.

The size or volume of data today exceeds petabytes and terabytes. Traditional methods of data storage and analysis can no longer keep up with the exponential growth and sheer volume of available data. Not only is velocity necessary for big data, but it is also necessary for all processes. Big data should be used as it streams into an organization so that its value can be maximized. This is especially important for time‐sensitive processes.

### 3.5. MapReduce

A very popular paradigm for parallel processing of large datasets over numerous nodes is known as MapReduce [33]. The MapReduce paradigm has now been adopted by NoSQL databases such as MongoDB [30] and open‐source frameworks such as Apache Hadoop [31]. The MapReduce framework deals with issues of communication, fault tolerance, parallelization, load balancing, and so on, automatically [32], so it is a much easier programming model to work with.

The MapReduce splits the data into smaller pieces and spawns copies of the programs on the cluster nodes, assigning a Map or Reduce task to each idle node when setting up. When we refer to a node being “assigned” to a Map task, we mean that it is assigned to perform the Map function. The Reduce node has similar definitions.

The Map function takes a (key, value) pair as input and produces a sequence of (key, value) pairs that are locally buffered on the Map node, as shown in Figure 2. The reduce nodes can then read the data from the buffers after they have received the coordinates, using remote procedure calls. Sorting all the intermediate values and grouping them according to intermediate key as (key, list of values): Reduce operation: Takes an intermediate key and list of values for that key and reduces them into a smaller list. This continues until none of the intermediate pairs remain to be dealt with. Intermediate values are given by an iterator in the case of Reduce. This means that we can handle value lists of a length that cannot be accommodated in RAM. The file that contains the results of the Reduce function is augmented with its output. To parallelize recursive algorithms, it may be necessary to perform multiple MapReduce phases. A computing node in a MapReduce process can receive more than one Map or Reduce task before the final Reduce step is done. This is why it is important to keep the outputs of Map and Reduce in the constrained format of (key, value) pairs.

**Figure 2:**
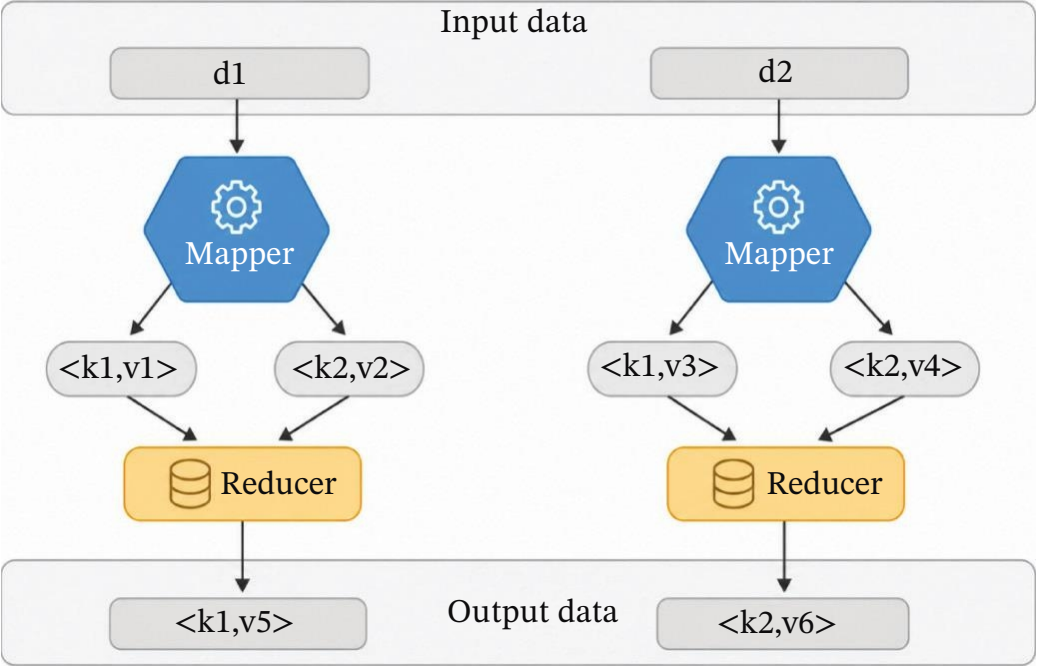
Baseline of Map and Reduce model.

A thorough knowledge of the MapReduce procedure is crucial to the creation of efficient MapReduce‐based algorithms for big data analysis. It is true that MapReduce enables parallel execution of the Map and Reduce tasks on different machines, but the output of the Reduce tasks is only available when they are complete [33]. This means that tasks for Reduce must wait until other Reduce tasks have completed before they can be released so that the node can be used for other tasks. The next MapReduce phase may not be invoked, as poor performance can be the result. To create efficient MapReduce‐based algorithms for big data analytics, it is helpful to have a good handle on the MapReduce process. Map and Reduce tasks can be executed in parallel on different machines, but the output of the Reduce task is only available when all of the Reduce tasks are completed. This means that once tasks have been completed on a node, the node will not be released for other tasks until other Reduce tasks have been completed. This will not only prevent the next iteration of MapReduce from being called, but it can also adversely affect performance.

### 3.6. Feature Selection (FS)

The term “feature selection” refers to the procedure of “discovering and discarding as much superfluous data as possible” [34]. To solve a problem, it is necessary to extract a set of features that adequately characterize the problem. There are additional benefits reported in the specialized literature [35] when using this subset when training a learner. To improve learning algorithms′ generalization abilities, FS can eliminate superfluous and redundant features that can cause accidental correlations. Overfitting is reduced when using FS, which is useful for later algorithms. As a result of FS, the search space established by the features will be smaller, which in turn will accelerate the learning process and require less storage space.

Because the use FS is useful even when the task at hand has nothing to do with the data mining algorithm used on the data, using FS during data collection can help reduce time, sampling, sensing, and personnel. When working with less complex data, models and visualizations become more comprehensible and interpretable.

## 4. The Proposed Framework of MO‐LSBCD

The primary objective of this paper is to address challenge of large‐scale data clustering, as existing data clustering approaches have proven to be inadequate in terms of time complexity and the capacity to extract relevant patterns from large datasets. The method developed in this study utilized the MapReduce architecture to handle large‐scale data. The optimal clustering process was begun by employing an optimization algorithm to produce the most optimal clusters. The modules inside the Optimized MapReduce Clustering Model (OMRCM) framework function based on an optimization method, with each module contributing to the achievement of optimal clustering. The mapper phase is inbuilt with a mapper function that follows the proposed BIRCH combined with SMA. To achieve the optimal centroids, certain measures must be performed. The reducer phase utilizes the generated centroids to formulate the best clusters, employing the MPA‐SMA method. The proposed methodology for clustering large‐scale data is aimed at reducing the time required for analyzing and extracting meaningful patterns from the data. Figure 3 depicts the block diagram demonstrating the proposed framework of MO‐LSBCD.

**Figure 3:**
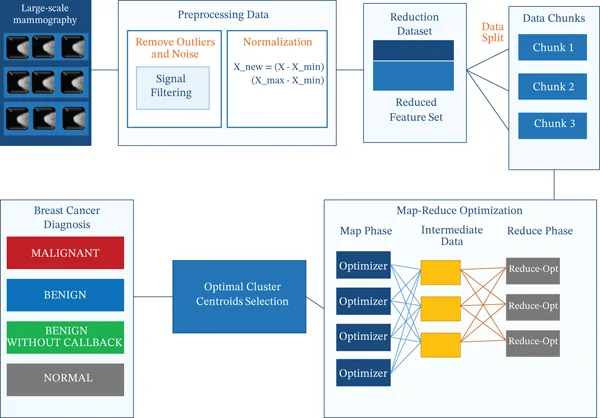
Architecture of the proposed framework (MO‐LSBCD).

The proposed framework of large‐scale data analytic (MO‐LSBCD) consists of 4 phases: (1) The first phase of framework depends on collected data; (2) preprocessing data that filters for unnecessary data; (3) proposing a new model to deal with large‐scale data according to optimization of MapReduce model; and (4) predicting breast cancer disease using deep learning classification. In the following, the stages of the new proposed framework of large‐scale data are discussed.

The proposed framework depends on MapReduce model (OMRCM) and improvements to the OMRCM′s clustering algorithms. Each mapper in the OMRCM makes use of a recently developed clustering algorithm called BIRCH‐SMA. This algorithm utilizes the SMA to determine the centers of each cluster. The reducers take the combined centroids from the mappers as input and use the proposed MPA‐SMA to find the best clusters for the data. To better estimate cluster centroids, the MPA‐SMA algorithm was developed by incorporating SMA into MPA. Figure 3 depicts a block diagram of the proposed MO‐LSBCD framework.

### 4.1. Dataset Collection and Preparation

The dataset used in this paper is CBIS‐DDSM, hosted on The Cancer Imaging Archive (TCIA) and accessed via Kaggle publicly [36]. CBIS‐DDSM is a contemporary, standardized version of the original DDSM (Digital Database for Screening Mammography), which comprised 2620 scanned‐film screening studies with verified normal, benign, and malignant pathology. CBIS‐DDSM was curated and standardized by [37] and summarized in Table 2 to overcome key limitations of the original DDSM, namely nonstandard proprietary image compression, imprecise region of interest (ROI) annotations, and the lack of a standardized evaluation protocol for computer‐aided detection and diagnosis (CAD) systems. As part of this curation, the original DDSM images were reviewed by a trained mammographer, losslessly decompressed and converted to DICOM format, normalized to standardized optical density values, and cropped/segmented at the ROI level. The Kaggle used in this work additionally converts these DICOM images to JPEG format while retaining the original resolution. For this paper, we further resized all images to 256 × 256 pixels, normalized pixel intensities to [0, 1], and applied ImageNet channel‐wise mean subtraction for compatibility with transfer learning. The complete data acquisition characteristics are summarized in Table 2 that reports 6775 studies/series; this reflects CBIS‐DDSM′s per‐abnormality case‐splitting structure, in which each patient is represented by multiple abnormality‐specific DICOM entries rather than 6775 distinct patients all entries derive from the same 1566 unique participants.

**Table 2:** CBIS‐DDSM dataset summary.

Property	Value
Full dataset name	Curated Breast Imaging Subset of DDSM
Imaging modality	Mammography (MG)
Body part examined	Breast
Number of studies	6775
Number of series	6775
Number of participants	1566
Total number of images	10,239
Image format (this work)	JPEG
Image storage size	~6 GB
Original DICOM size	~163 GB
Data license	CC BY‐SA 3.0
Repository	Kaggle

The unit of analysis for the classification task discussed in this paper is not the raw image file, but rather the annotated abnormality (ROI). The number of images in the dataset is 3568 ROI‐level records from 1566 patients; the higher number of images in Table 2 10,239 is due to the multiple images associated with each abnormality (full mammogram, ROI mask, and cropped patch) that are not used separately as distinct classification samples in this paper. Individual records are a mammographic ROI associated with an abnormality detected. The dataset is 3568 annotated records of 1566 distinct patients. Individual records are a mammographic image of an area of interest (ROI) related to a detected abnormality. The data is divided into two different subdatasets depending on the nature of the abnormality: calcification and mass. The subdatasets are further broken down into training and testing partitions as summed in Table 3.

**Table 3:** Distribution of dataset.

Subdataset	Training set	Test set	Total
Calcification	1546	326	1872
Mass	1318	378	1696
Total	2864	704	3568

The most important attribute to predict is the pathology attribute that is the histologically confirmed diagnosis of each abnormality. It has three class labels: Malignant: Tissue that has been diagnosed to be cancerous, and which needs urgent clinical treatment. NORMAL: Tissue that does not contain any cancer cells. BENIGN IN WITH CALLBACK: Noncancerous tissue that was adequately suspicious for further imaging. The data show a close balance between the number of malignant and benign cases, which is good for training unbiased classification models. For binary classification experiments, the BENIG and BENIG_WITHOUT_CALLBACK classes are grouped together into a single class, BENIG, with 1457 malignant cases (40.8%) and 2111 benign cases (59.2%). Table 4 shows the distribution of classes on the entire dataset.

**Table 4:** Class distribution of the target variable (pathology).

Class label	Count	Percentage (%)
MALIGNANT	1,457	40.8%
BENIGN	1429	40.1%
BENIGN_WITHOUT_CALLBACK	682	19.1%
Total	3568	100%

Figure 4 displays the range of distributions of the CBIS‐DDSM cancer diseases dataset in all its subsets and pathology classes. The pathology counts displayed in Panel (a) as a breakdown of the counts by the type of abnormality (calcification and mass) and the division of the data (training and test) shows that the majority of the BENIGN_WITHOUT_CALLBACK cases (474) are attributed to the calcification training subset, whereas the majority of the MALIGNANT cases (637) are credited to the mass training subset. In Panel (b), the overall class distribution across the entire dataset (3568 records) is shown, which is a near‐balanced binary structure between malignant cases (MALIGNANT: 40.8%) and combined benign cases (BENIGN: 40.1; BENIGN_Without_Callback: 19.1%). This equal distribution is a crucial quality to train unbiased deep learning classifiers, as this lowers the risk of model bias towards any given pathology class and ensures reliable assessment of the proposed MO‐LSBCD framework.

**Figure 4:**
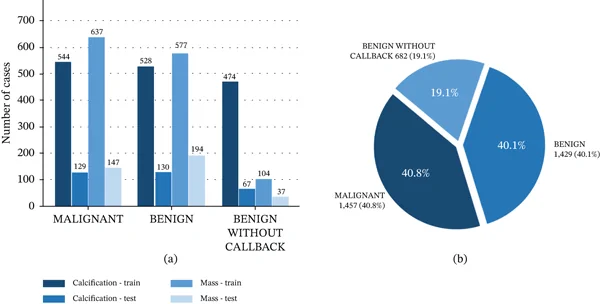
Distribution variety of breast cancer diseases dataset. (a) Pathology distribution by subset. (b) Overall class distribution (*N* = 3568).

### 4.2. Preprocessing Data

Data preprocessing consists of removing outliers, normalization, and dimensionality reduction:

• Removing outliers: The statistical power and quality of the trained model are both enhanced by the removal of outliers because of the reduction in data variability. The local outlier factor, an unsupervised anomaly detection method, was used to identify statistical outliers. A data point′s local density deviation relative to its neighbors is computed. This means that an outlier sample [38] is one that has a significantly lower density than its surrounding samples.
• Feature normalization **[** 39]: By adjusting their relative sizes, features undergo a process called “normalization.” The model′s efficiency and consistency in training are both improved as a result. A feature x, for instance, was linearly scaled from 0 to 1 as in

(1) xi=xi−MinMax−Min,

where Min and Max represent the lowest and highest possible values for that characteristic.

• Dimensionality reduction: Using this technique, we learn a method for cutting down on the features required to process an entire dataset. Adding more features to a model usually makes the task of prediction more difficult. Removing features with low variance is a straightforward method for dimensionality reduction [40] that was employed here.

### 4.3. Proposed of OMRCM

This section presents the proposed framework for large‐scale data analytic using the Optimized MapReduce Clustering Model (OMRCM), which consists of two proposed methodologies and first method is called BIRCH‐SMA that used for large‐scale data clustering adopted in the OMRCM. The OMRCM framework applies a unique clustering technique in each mapper, wherein the cluster centroids are determined through the utilization of BIRCH‐SMA optimization. The centroids acquired through the mapping process are combined and utilized as input for the reducing phase that is considered the second method inside the proposed framework called the MPA‐SMA, which is utilized to identify the best clusters for data clustering. The MPA‐SMA technique is utilized in clustering for calculating the centroids for the final cluster. It is created by integrating SMA into MPA. The proposed MapReduce optimization approach for large‐scale data clustering is depicted in block form in Figure 5.

**Figure 5:**
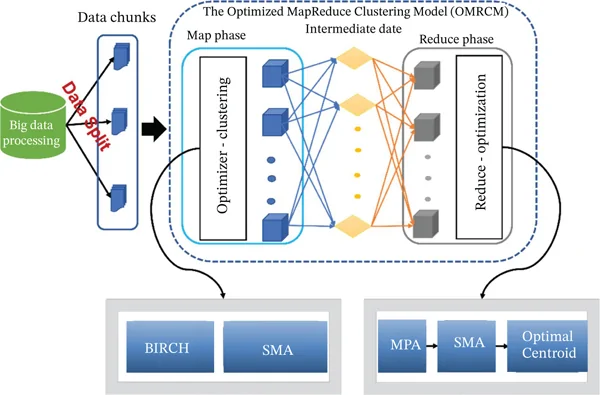
The MapReduce model optimization.

This subsection discusses the proposed OMRCM model, which is tailored to clustering large amounts of data. OMRCM is a programming model that includes mappers and reducers. It is a clustering algorithm that works by simultaneously processing multiple copies of the data partitions. Thus, the MapReduce model can be used to manage massive datasets by dividing up a single task across multiple nodes in a cluster. The mappers in OMRCM take the partitioned data as input and use the BIRCH‐SMA clustering optimization algorithm to determine where the clustering centroids should be placed. During this time, the MPA‐SMA algorithm is utilized by the reducer functions to pick the best cluster centers possible. Assume DB is considered the database, and assume it contains m records, each with n attributes, as depicted below.

(2) B=dk,l;00≤k≤m;≤l≤n.

Data in the database is denoted by d_*k*,*l*_, where *l* is the number of mappers in the OMRCM, and *k* is the number of partitions in the database. The following equation depicts the data partitions:

(3) bk,l=Dq;1≤q≤NM,

where NM refers to the overall number of mappers in the OMRCM as follows:

(4) M=M1,M2,⋯,Mp,⋯,MNM;1≤p≤N.

Hence, the input to the *p*th mapper can be given as

(5) Dp=dr,l;11≤r≤mp;≤l≤n,

where d_*r*,*l*_ represents the number of partitions in the data given to the *p*th mapper, and m_*p*_ represents the total amount of data in the mapper. Every mapper generates intermediate results by mapping its input data to some number of user‐defined clusters. As a result, the cluster center is represented by the result of each mapper that is context‐ and data‐dependent. All NM mappers produce output of size m × n, shown here as follows:

(6) I=p1p2⋯pN,

where *p* is the result of the mapper and stands for the center of the cluster. The mapper′s output is fed into a group of reducers, which receive it as input in the form that represents as

(7) R=R1,R2,⋯,Ru,⋯RNR;1≤u≤NR,

The number of reducers is denoted by NR. To create the final clusters, the reducers combine the centroids of the clusters obtained from the mappers, as shown below:

(8)  Cz=cx,lz;1≤x≤Cz1≤l≤n1≤z≤P.

*C*_*z*_ is the cluster size in the *z*th reducer, and $c_{x,l}^{z}$ is the cluster in that reducer.

The following demonstrates two methods (Clusters centroid and Select Optimal Clusters Centroid), which used to optimize the MapReduce framework.

The MPA component of our hybrid optimization established guidelines from the swarm intelligence literature, with adaptations for our specific application domain. Table 5 presents the configuration.

**Table 5:** Marine Predators Algorithm parameters.

Parameter	Value
Population size/number of search agents (NN)	50
Maximum number of iterations (*T*_Max_)	200–500
Fish aggregating devices probability (FADs)	0.2
Movement scaling parameter (P)	0.5
Lévy distribution parameter (*β*)	1.5
Maximum iterations	500

The distributed computing configuration was determined based on the characteristics of our experimental infrastructure and dataset properties. Table 6 details the MapReduce architecture parameters.

**Table 6:** MapReduce configuration.

Parameter	Value
Number of mapper nodes	8
Number of reducer nodes	4
Images per partition	9886
Data replication factor	2
Combiner function	Weighted average

#### 4.3.1. Optimized BIRCH‐Based Centroid Estimation Using Mapper Functions

In this phase, we introduce a new approach to optimizing clusters using the (BIRCH) combined with SMA, which is generated (BIRCH‐SMA). The BIRCH is used to tweak SMA that searches for cluster centers. Centers are estimated by the proposed fractional SMA, which uses systematic initialization itself. The parameters used in the proposed method are not problem‐specific and thus make the technique very robust. Using the BIRCH SMA, we are able to optimize the centroid of our clusters and complete the clustering procedure. By iterating until the tolerance is met, the optimal solution points for the center are chosen. This allows for an optimized clustering process to be completed.

The clustering algorithm is initially fed the datasets it will be grouped into as input. Object and attribute distinctions are used to filter the collected data. Based on that attribute, *x* *j*1 is the object in issue (consideration). Here, *j* represents the criterion by which the object is chosen. After that, the clustering algorithm is applied to the data from the chosen objects. Here, we employ the fractional SMA algorithm as our clustering mechanism of choice. Solution point initiation is required prior to solution point estimation in a process.

The solution point is used to guide which vectors are crossed over and mutated. The solution vector points are crossed using a single point crossover with dual probability. Clustering range vectors are swapped point‐for‐point to accomplish the crossover. The grouping reveals alternative trajectories for solving the problem. Mutation occurs at the crossover points where the solutions are found. Because of the mutation, the coordinates for the new solution are now known. Fast mutation is used to obtain the vectors of the solutions. After solution vectors are obtained, the clustering procedure uses a fitness evaluation to decide which solution points to demonstrate. The methods employed here are strikingly similar to the SMA. The mutation is locked in the new state permanently.

Using SMA, BIRCH is tweaked. In addition to the solution point, the mutation and crossover processes produce new solution vectors, which serve as the solution points. Within the given range, if the fitness value of the randomized solution vector is higher, the solution point will replace the random point. The centroid points are the solution points that are being referred to here. The best optimal centroid for the clustering is then selected using the fitness function from the computed solution points, and the process is repeated until the tolerance is met.

Grouping is performed after the centroid point, or optimal centroid point, is estimated. Due to the availability of the bulk solution point, the proposed algorithm facilitates quick centroid estimation. Thus, the proposed algorithm will be helpful for data clustering with large datasets, varied scale, etc. If the fitness value is greater than the initiated solution point.

##### 4.3.1.1. The Proposed Hybrid Method of BIRCH and SMA

This paper presents an effective hybrid clustering algorithm called BIRCH‐SMA, which combines the merits of the BIRCH algorithm and SMA. The BIRCH component can build small cluster summaries and form candidate cluster centroids for large datasets. The optimal centroids are then optimized using the SMA component by searching the search space and choosing the optimal cluster centers. Hence, BIRCH‐SMA is a two‐stage hybrid optimization strategy: BIRCH is used to efficiently perform hierarchical clustering and summarization, and the centroid positions are refined by SMA to enhance the overall quality of the clustering results.

Suppose there are *n* objects in the dataset, *c* is a specified number of clusters, vi is the centroid vector of the *i*th cluster, and xi (*t*) is the position of the *i*th candidate solution at iteration *t*. The goal of the optimization process is to change the centroid vectors so that the clustering objective is minimized and convergence is reached. In the hybrid system the main variables are defined as follows:

*n* is number of objects in the dataset, *c* is the number of clusters that are already specified, vi is the centroid vector of the *i*th cluster, xi (*t*) is the position of the candidate solution at generation *t*, and uij is the membership degree of object *i* in cluster *j*.

In the BIRCH‐SMA model, the BIRCH‐generated centroid vectors are considered as potential solutions to the SMA optimization stage. The SMA search mechanism is used to update these centroid vectors in an iterative fashion until the optimal position of the centroid vectors is obtained. This enables the algorithm to retain the advantages of BIRCH in terms of efficiency for processing large‐scale data and improve the quality of the centroid by exploiting the global optimization ability of SMA.

Several parameters are important to the BIRCH algorithm that affect the quality of the clusters. The BIRCH configuration is summarized in Table 7, and the SMA parameters used for optimization of the centroid are shown in Table 8. These parameters were chosen to optimize clustering efficiency, stability of convergence and search exploration.

**Table 7:** BIRCH parameters.

Parameter	Value
Number of clusters (*c*)	88
Branching factor (*B*)	50
Threshold (*T*)	0.5
Maximum iterations	500
Distance metric	Euclidean

**Table 8:** Slime mould algorithm parameters.

Parameter	Value
Population size (*N*)	50
Maximum iterations (*T*)	500
Visit table dimensions	*N* × *N*
Guided foraging probability	0.9
Territorial foraging probability	0.1
Migration coefficient	0.5

In this section, we propose that using BIRCH as an initial centroid search is a good approach to enhance the performance of the SMA optimization stage. BIRCH offers compact and stable cluster summaries that limit the search space and enhance the quality of the initial centroid candidates. The SMA algorithm then conducts an adaptive global search among these candidate centroids to find the best final clustering solution. This hybrid approach is especially beneficial for large datasets, as it merges the advantages of hierarchical clustering with metaheuristic optimization.

BIRCH‐SMA optimization is a two‐stage process, based on the clustering objective function. The first stage is to build a CF‐tree by BIRCH and retrieve the centroid candidates from the leaf entries. In the second stage, SMA iteratively updates the candidate centroids until convergence is achieved. The proposed model is thus a scalable and effective solution for data clustering at large‐scale in pseudo‐code 1:

**Pseudo-Code 1: BIRCH‐SMA**
Input:
   •Dataset D
   •Number of clusters c
   •BIRCH parameters
   •SMA parameters N, T, W
   •Maximum iterations MaxIter
Output:
   •Final cluster centroids
   •Final clustering result
1. Initialize the dataset D and set iteration counter t = 0.
2. Build the BIRCH CF‐tree using the input dataset D.
3. Extract candidate centroid vectors from the CF‐tree leaf entries.
4. Initialize the SMA population with the candidate centroids.
5. While t < Max_Iter_ do
6.     For each candidate solution xi do
7.         Compute the clustering fitness value using the objective function.
8.         Update the candidate centroid position xi(t+1) using the SMA search rule.
9.     End for
10.    Update the centroid vectors vi(t+1).
11.    Check convergence condition.
12.    If convergence is satisfied, break.
13.    t = t + 1
14. End while
15. Return the best centroid vectors and final clusters.

#### 4.3.2. The Integration Mechanism Between BIRCH‐SMA Clustering Phase and the Classification

The integration between the BIRCH‐SMA clustering phase and the classification phase in the proposed MO‐LSBCD framework operates through three complementary mechanisms: (1) feature augmentation, (2) cluster‐based sample routing, and (3) MapReduce distribution strategy, as described below.

##### 4.3.2.1. Mechanism 1: Feature Augmentation

The vectors generated by BIRCH‐SMA clustering serve as additional input features for the classification models. For each image *x*_*i*_, the clustering phase produces a membership vector:

μi=μi12,μi,⋯,μic,

where *c* is the number of clusters and *μ*_ij represents the degree of membership of sample *i* to cluster *j*. These membership values are concatenated with the CNN‐extracted feature vector f (x_i_):

x′i=fCNNxi μi12,μi,⋯,μic.

This augmented feature vector $x_{i}^{\prime }$ is then fed to the classifier, providing both visual features and cluster‐context information. Figure 6 depicts feature augmentation process.

**Figure 6:**
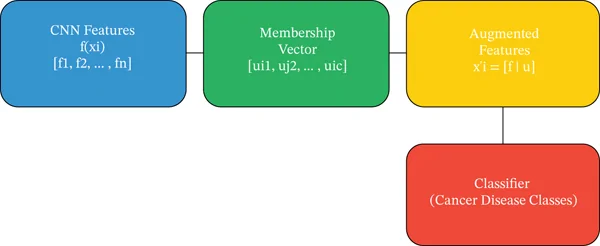
Process of feature augmentation.

##### 4.3.2.2. Mechanism 2: Cluster‐Based Sample Routing

The cluster assignments from BIRCH‐SMA determine routing decisions for samples to specialized subclassifiers. Each cluster c_k_ groups samples with similar characteristics, which allows the system to

• Route malignant mammogram images to a classifier specialized in detecting MALIGNANT disease.
• Route benign mammogram images to a classifier specialized in recognizing BENIGN disease.
• Route low‐suspicion images to a classifier specialized in BENIGN_WITHOUT_CALLBACK cases.

The routing function R is defined as follows:

Rxi=argmaxkμik⟶Classifierk.

This enables each subclassifier to focus on a smaller, more homogeneous subset of classes, improving classification accuracy. Figure 7 clarifies cluster‐based sample routing.

**Figure 7:**
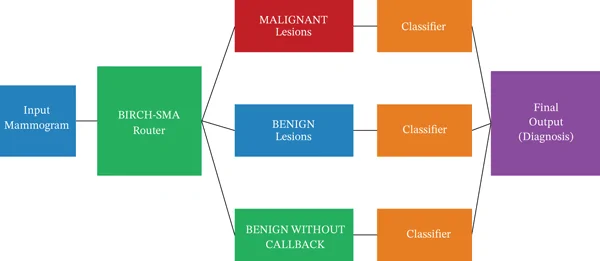
Sample of cluster‐based routing.

##### 4.3.2.3. Mechanism 3: Strategy Distribution of MapReduce

In the distributed computing context, cluster assignments determine how data is partitioned across MapReduce nodes for parallel processing:

• Map phase: Cluster membership determines which mapper node processes each sample
• Shuffle phase: Samples are grouped by cluster‐class combinations
• Reduce phase: Aggregation of classification results based on cluster context

This strategy ensures that similar samples (same cluster) are processed together, improving computational efficiency and enabling parallel classification. Figure 8 depicts MapReduce distribution strategy.

**Figure 8:**
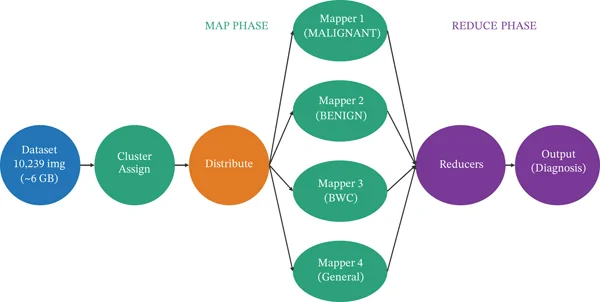
Strategy of MapReduce distribution.

#### 4.3.3. Optimal Cluster Selection via MPA‐SMA With Previous‐Phase Centroid Estimation

This section presents the utilization of the MPA technique in conjunction with the SMA, referred to as the MPA‐SMA algorithm, for the purpose of picking the most optimal clusters during the reducer phase in large data clustering. The optimization of the reducer function can be achieved through the utilization of a hybrid method known as Marine Predators Algorithm and artificial hummingbird method (MPA‐SMA). The MPA‐SMA algorithm is formulated by integrating the SMA [27] and MPA algorithm [28], where the update rule of the MPA algorithm is adapted by incorporating the update rule of the SMA algorithm. The MPA‐SMA algorithm combines the strengths of both MPA and SMA, resulting in a balanced integration of their respective advantages and disadvantages. The hunting mechanism of the hummingbird effectively maintains equilibrium during both the exploration and exploitation stages. The effectiveness of SMA in addressing real‐time challenges and its ability to function in unfamiliar search spaces have been demonstrated. The algorithm demonstrates distinct characteristics by utilizing either random or optimal search strategies within the solution space to pursue the prey. The solution parameters are effectively situated within three distinct search modes, namely the encircling phase, the exploring phase, and the exploitation phase. By adapting MPA′s update procedure with SMA, MPA‐SMA improves its ability to perform the clustering task. To determine the cluster centroids, a reducer combines the local centroids produced by the mappers. The intermediate results from the mappers are used by the reducer, which is defined as

(9) I=Iy,l;11≤y≤NC;≤l≤n.

The overall of mappers denoted by *N* and the overall of clusters denoted by *C* so for each mapper, the number of clusters denoted by *N*_*C*_, whereas *I* ^*y*,*l*^ is the intermediate output from *N*. Reducers are responsible for locating clusters by applying the following definitions to the data *I* obtained from mappers:

(10) RI=ck.

The total number of clusters denoted by *c*_*k*_ in the *k*th reducer. As a result, the reducer produces the result of the following function:

(11) RP=Pk,l;1≤q≤N 1≤k≤NmC1≤l≤N.

The number of clusters denoted by the variable $N_{m}^{C}$ in the *q*th mapper. The calculation is performed with the MPA‐SMA method introduced in this paper, which is aimed at constructing the most optimal cluster centroids for the clustering process. The fitness of each cluster is determined based on the Davies–Bouldin index (DB‐index), a commonly used metric in clustering evaluation. Therefore, with the MPA‐SMA method, the reducer effectively organizes the data into clusters, with the specific number of clusters being determined by the user. MPA is a stochastic optimization technique that emulates the collective behavior observed in fish schooling. On the other hand, the SMA is a nature‐inspired algorithm that has been devised by drawing inspiration from the behavior of hummingbirds. By including SMA into the update process of the MPA algorithm, it is possible to enhance the performance of the proposed method in the context of clustering. The reducer uses the Marine Predators Algorithm with adaptive hybrid algorithm (MPA‐SMA) to process the data, which is represented as vectors, to identify clusters.

#### 4.3.4. Fitness Function Representation

Clustering accuracy, DB‐index [41] and Dunn index (Dunn‐Index) [42] are used to measure the efficiency of the proposed approach to clustering big data. The DB‐index is calculated as in Equation (12), and the classification precision relies on the total number of categories. The optimal strategy provides the highest possible classification accuracy and DB‐index.

Finding the best clusters with the proposed model, Optimized MapReduce Clustering Model (OMRCM) is represented most simply through the solution encoding. In this case, the cluster centroid serves as the solution, and it is randomly initialized based on the intermediate data generated by the mappers. This means that the solution is a vector, with a length proportional to the number of clusters and the data. The MPA‐SMA algorithm allows for optimal determination of cluster centroids based on fitness assessed via the DB‐index.

The quality of the solution is determined by the fitness function, which is implemented in the form of a DB‐index [42] like that used in [43]. To determine how similar two clusters are to one another, the DB‐index can be used. This index works well with distance‐based clustering algorithms. Based on the DB‐index, the fitness function is formulated as follows:

(12) DB=1ci ∑e=1ciFe.

*F*_*e*_ represents the function for choosing the maximum value of the similarity metric between the clusters according to the following equation:

(13) Fe=MaxSe,f,f≠e.

The similarity measure represented via *S*_*e*,*f*_ that calculates the degree of proximity between clusters using their respective Euclidean distances and is depicted as follows:

(14) Se,f=Ae+AfEe,f.

The scattering measures between two clusters depicted by *A*_*e*_ and *A*_*f*_. The Euclidean distance between the two cluster matrices represented by *E*_*e*,*f*_. Clustering performs better when there is less space between the cluster center and the data points.

(15) Ee,f= Le−Lf .

The two clusters that generated from the centroids depicted by *L*_*e*_ and *L*_*f*_. Distance from the cluster to the input data used to determine *A*_*e*_.

(16) Ae=1me ∑e=1meIy,l−Le.

The number of data points is represented by *m* ^*e*^ related to *L*. For optimal clustering, the distance between any two centroids should be maximized, even though it is essential that the distance between any two centroids be minimized.

The distance to the centers of the points in the data must be as small as possible; the distance between two centroids must be as large as possible for better clustering.

The second metric evaluation is Dunn‐Index represented by

(17) Dunn=MinjMink,k≠j dvj,vkMax Δvj.

Construction of a validation standard: A larger Dunn‐Index is obtained when clusters are more widely separated, and the DB value is lower when similarities within clusters and differences between clusters are high.

(18) Clusteraccuracy=1m ∑k=1NcMaxj=1cLci∩cil.

The total number of observations is denoted by *m*, the number of clusters is indicated by *N* ^*c*^, the number of classes is denoted by *c* ^*L*^, the *i*th cluster is denoted by *c*_*i*_, and the *i*th class is denoted by $c_{i}^{l}$.

### 4.4. Proposed Prediction of Breast Cancer Diseases

The process used for identifying diseases in breast cancer is illustrated in Figure 9. In this section, we will clarify the individual components of the proposal, outlining each step comprehensively.

**Figure 9:**
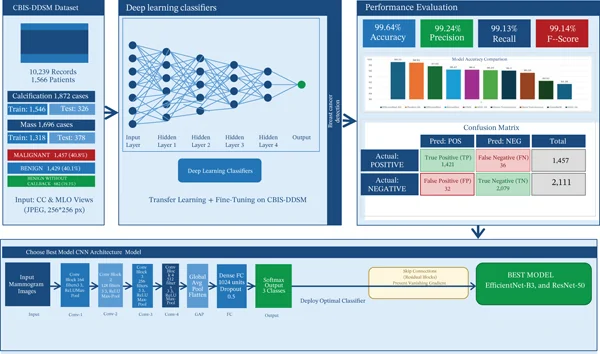
The breast cancer disease detection model.

The proposed framework begins by gathering input mammographic images that will represent a wide range of breast tissue abnormalities, including the type of calcification (amorphous, coarse heterogeneous, fine linear, fine pleomorphic, and vascular), the mass shape (irregular, lobular, oval, and round), and the mass margin characteristics (circumscribed, ill‐defined, microlobulated, obscured, and spiculated) as found across the CBIS‐DDSM dataset. These raw radiological images are acquired using the standard clinical digital mammography systems and both craniocaudal (CC) and mediolateral oblique (MLO) views are acquired on each patient. All images during the experimental and preparatory steps of the framework are converted to standardized representations of JPEG and resized to 256 × 256 pixels so that they can be compatible with the deep learning classifiers.

The dataset may consist of unprocessed images that could contain noise, so they must be preprocessed before being fitted into the learning module. In the preprocessing stage, image enhancement methods such as rotation, scaling, and shearing are employed to prepare the image for further analysis.

We use the following steps to create the predictive model:

• Images are being extracted from the dataset.
• Image data should be preprocessed by resizing and rotating images.
• Converting convoluted features as fully connected (FC) layers.
• The data are flattened and transformed into a one‐dimensional array or vector, which is subsequently connected to one or more FC layers.

In the final step, the features associated with the various types of input are retrieved. The proposed model′s detailed architecture is depicted in Figure 9.

In this section uses the deep different learning methods in the proposed framework. The VGG‐16, also known as the VGG Net‐16, is a convolutional neural network (CNN) architecture that consists of 16 layers. It is widely recognized and frequently employed in the field of image classification for identifying diseases due to its uniform design and vast utilization. One of the main contributions of VGG‐16 is in its demonstration that, under certain conditions, increasing the intensity of the network can enhance overall system performance [44]. The VGG‐16 transfer learning method comprises three key components, namely convolution layers, FC layers, and pooling layers. The Convolutional layer, commonly referred to as Conv, is a fundamental component in deep learning models that applies filters to images to extract relevant information.

VGG‐19 transfer learning model was the CNN which was initially introduced in [45]. The model consists of three densely connected layers, 16 convolutional layers, and 19 layers for the purpose of classifying images into 1000 distinct categories. The model consists of three FC layers, two convolutional layers followed by one max pooling operation, four convolutional layers followed by one max pooling operation, and two conv layers subsequent to one max pooling operation. The prediction of image model is widely recognized for its popularity, mostly due to the extensive utilization of 3 × 3 filters in each ConvNet [46].

The ResNet family includes the ResNet‐50 model [47]. There are 48 convolutional layers in the ResNet‐50 model in addition to a MaxPool and an Average Pool layer, forming its architectural composition. The ResNet model is widely acclaimed for its efficacy in image categorization. The ResNet‐50 architecture consists of four primary stages. The initial convolution stage is composed of three layers: a 1 × 1 kernel with 64 channels, a 3 × 3 kernel with 64 channels, and lastly a 1 × 1 kernel with 256 channels. At this stage, the three layers have undergone a process of replication three times, resulting in the existence of a cumulative total of nine layers. Subsequently, an initial convolutional kernel of size 1 × 1 with 128 channels is seen, followed by a subsequent convolutional kernel of size 3 × 3 with 128 channels, and finally, a convolutional kernel of size 1 × 1 with 512 channels. This pattern is repeated six times, resulting in a cumulative total of 18 layers. Subsequently, a kernel with dimensions of 1 × 1 and 512 channels was created, which was followed by the construction of two additional kernels with dimensions of 3 × 3 and 512 channels, and 1 × 1 and 2048 channels, respectively.

EfficientNet [48] proposes compound scaling: scaling the depth, width, and input resolution of the network simultaneously according to a single principle rather than tuning each dimension individually. This synchronized scaling enhances the accuracy per compute, making it suitable for large medical imaging workloads that demand performance and efficiency.

EfficientNet‐B3 [48] is a larger version of EfficientNet, using the same compound‐scaling concept, but with a greater depth/width/resolution than smaller options. It contains more detailed lesion patterns and finer texture details, which is why it has achieved high classification accuracy in your mammography experiments.

DenseNet [49] has dense connections; that is, each layer is connected to all the previous layers, which promotes feature reuse and gradient propagation. This design results in less repetition of learning, more training stability, and can be effective in situations where fine details in the visual information (such as mass boundaries) need to be maintained.

The Vision Transformer (ViT) [50] model considers an image as a series of patches and uses self‐attention to capture the global relationships across the entire image. It can capture long‐range dependencies between distant tissue regions, which is beneficial for analyzing complex breast patterns; however, it generally requires large amounts of data and careful tuning.

Swin Transformer [51] fuses transformer attention with computational locality with shifted‐window self‐attention and a hierarchical feature pyramid. It maintains high level of expressiveness of the transformer while keeping costs low, providing a balance for high‐resolution medical images and structured lesion localization.

ConvNeXt [52] is a reimagining of convolutional networks for the transformer era, featuring CNN blocks designed for training and architecture with transformer style (e.g., larger kernel and more sophisticated normalization/activation strategy). It maintains the CNN robustness and deployment simplicity and closes the performance gap between transformers, making it a good practical baseline in clinical pipelines.

## 5. Performance Evaluation Metrics

The performance measures of classification are accuracy, sensitivity, precision, F1‐score, and the Youden index, which are derived from values TP, TN, FP, and FN of a confusion matrix in Figure 10. The corresponding mathematical formulations of these evaluation measures are provided in Equations (19)–(24). In addition to these measures, AUC, F‐value, and G‐value are also employed to provide a more comprehensive assessment of classification performance.*F* *G* Suppose the data are separated into two categories, positive and negative, in the confusion matrix.

**Figure 10:**
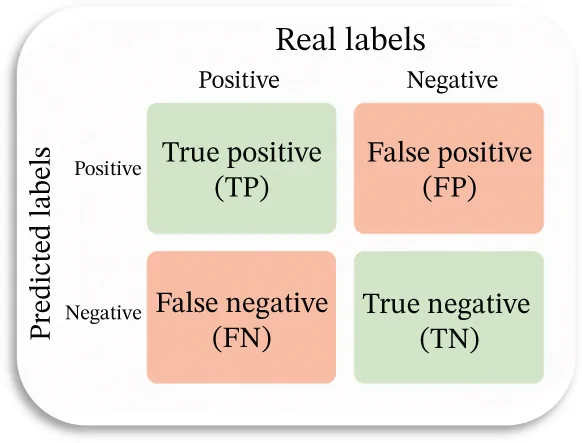
Confusion matrix for performance.

Given the specific requirements of breast disease classification—including multiclass categorization (3 disease classes), we prioritize evaluation metrics based on their relevance to the problem domain, as summarized in Table 9 and for the multiclass breast disease classification problem comprising three disease categories, we employ weighted averaging for computing precision, recall (sensitivity), and F1‐score metrics. The weighted averaging approach is specifically chosen for the following reasons:

**Table 9:** Evaluation metrics prioritization for breast cancer disease classification.

Metric	Priority	Averaging method	Average method formula
Recall	Highest	Weighted	*Σ*(*n*_*i*_ × Recall_i_)/*Σ* *n*_*i*_.
F1‐score	High	Weighted	*Σ*(*n*_*i*_ × F1_*i*_)/*Σ*n_i_.
Precision	Moderate	Weighted	*Σ*(*n*_*i*_ × Precision_i_)/*Σ* *n*_*i*_.
Specificity	Moderate	Weighted	Specificity*_i_*.
AUC	Moderate	Weighted	*Σ*(*n*_*i*_ × AUC_*i*_)/*Σ*n_i_.
*G*‐value	Supplementary	Weighted	*Σ*(*n*_*i*_ × G_*i*_)/*Σ* *n*_*i*_.
Accuracy	Reference	Micro (overall)	*Σ*TP_*i*_/total samples.

Recall (sensitivity) is prioritized as the most critical metric because undetected breast cancer diseases (false negatives) have severe consequences in settings. Infected breast cancer that goes unidentified can spread pathogens to breast, resulting in widespread economic losses. Therefore, the proposed system emphasizes maximizing recall to ensure comprehensive disease detection, even at the cost of occasional false positives.

Specificity measures the proportion of actual negative (healthy) samples correctly identified by the model. High specificity indicates the model′s ability to correctly identify healthy breast tissue, reducing false disease diagnoses that could trigger unnecessary treatment interventions.

F1‐score is emphasized due to the inherent class imbalance in the breast cancer disease classification dataset. Since sample counts vary significantly across 88 disease categories, metrics that weight all classes equally may mask poor performance on minority disease classes. The F1‐score, as the harmonic mean of precision and recall, provides equitable assessment across all categories.

Precision is prioritized at a moderate level to balance false alarm reduction. In precision, false positive predictions could lead to unnecessary chemical treatments, increasing operational costs and environmental impact. However, in disease detection scenarios, the cost of missing an infection generally outweighs the cost of false alarms.

Accuracy is reported for comparability with existing literature but is interpreted cautiously. With 88 classes of varying sizes, overall accuracy may not reflect true model performance on underrepresented disease categories.

The mathematical formulations of these evaluation measures are defined as follows:

(19) Specificity=TNTN+FP,

(20) G−value=TPTP+FN X TNFP+TN, 

(21) Precision=TPTP+FP,

(22) Recall=TPTP+FN,

(23) F1−score=2∗precision∗recallprecision+recall,

(24) Accuracy=TP+TNTP+TN+FP+FN×100.

For the multiclass classification of breast cancer disease categories, precision, recall, and F1‐score are computed using weighted averaging, where each class metric is weighted by its support (number of true instances). This approach accounts for the dataset (ranging from 79 to 11,390 samples per class) and provides performance estimates reflective of actual disease distribution in settings. Overall Accuracy is reported as the microaveraged metric (ratio of correctly classified samples to total samples). AUC is computed using the One‐versus‐Rest (OvR) strategy with weighted averaging across all disease classes.

## 6. Experiments and Discussion

This section discusses the results of the tests, as well as the stages that were investigated and the proposed framework. This same machine, configured according to the parameters shown in Table 10, is used to run all of the outcomes. MATLAB was used for all of the experiments, and it was also used to extract and analyze the results. This section discusses the large data clustering method′s outcomes and analysis in order to demonstrate the method′s efficiency. Experiments results divided into three parts, and they are as follows: (i) experiments for estimate centroid in map phase using proposed BIRCH‐SMA and selection optimal centroid in reducer phase, and (ii) performance evaluation for classification to detect early breast cancer diseases.

**Table 10:** The specification details settings.

Specification name	Specification settings
SW specification	
OS	Windows 10
Programming language	MATLAB R2019a
HD specification	
Processor	Core(TM) i7‐4500
Frequency	2.40 GHz
RAM	16G
Hard disk	1 TB

To evaluate the effectiveness of the proposed framework, we conducted experiments on breast cancer dataset.

We compared the performance of our proposed framework with two other state‐of‐the‐art data analytics algorithms: The proposed framework achieved higher accuracy, precision, recall, and F1‐score in experiments than any of the competing techniques.

### 6.1. Image Preprocessing and Input Specifications

All the input images are resized to a size of 256 × 256 pixels to be consistent with the pretrained architectures of the models used in this work. Pixel intensity values are normalized in the range [0, 1] by dividing them by 255. Channel‐wise mean subtraction is performed using the statistics for the ImageNet dataset for transfer learning models.

### 6.2. Training Protocol

The data were split into the training, validation, and testing sets using the stratified sampling approach, with the proportion of data allocated as 70%, 15%, and 15%, respectively. The random number generator was set with a predetermined seed number, that is, 42, using the MATLAB function rng() for reproducibility of the experiments. The training process was carried out using the Deep Learning Toolbox in MATLAB, with early stopping set as patience equal to 10, preventing the model from overfitting during the training process. The experiments were performed using the NVIDIA RTX 3080 GPU with 10 GB of VRAM.

### 6.3. Model Specific Configurations

For the transfer learning experiment conducted with various deep learning classifiers, the convolutional layers were first frozen for 5 epochs and the classification head was trained. Then all the layers were unfrozen and fine‐tuned for an additional 20–50 epochs, with a learning rate of 1e‐5. Table 11 summarizes the batch configuration used for both the head‐training and fine‐tuning phases. And also, the complete two‐phase learning rate schedule is summarized in Table 12, which reports the full two‐phase learning rate schedule. A higher initial learning rate is used to quickly train the new classification head, whereas the pretrained convolutional base is frozen, followed by a much lower, piecewise‐decayed learning rate once all layers are unfrozen to avoid destructive updates to the pretrained weights.

**Table 11:** The batch size and related training settings based on GPU memory.

Parameter	Value
Minibatch size	32
Shuffle frequency	Every epoch
Input image resolution	256 × 256 × 3
GPU memory used for training	NVIDIA RTX 3080, 10 GB VRAM

**Table 12:** Two‐phase learning rate schedule used for transfer learning.

Phase	Epochs	Initial LR	Schedule type	Drop factor	Drop period
Phase 1: Head training (base frozen)	5	1e‐3	Piecewise	0.5	Every 2 epochs
Phase 2: Fine‐tuning (all layers unfrozen)	20–50	1e‐5	Piecewise	0.1	Every 10 epochs

### 6.4. Experiments of Proposed MapReduce Optimization Phase

The real‐world and artificial datasets are used in the study′s experiments. Figure 11 depicts visualization clusters of data points after using proposed BIRCH‐SMA. Figures 12 and 13 show the results of using various algorithms. Since BIRCH‐SMA has the highest Dunn‐Index and the lowest DB‐index, it produces superior results.

**Figure 11:**
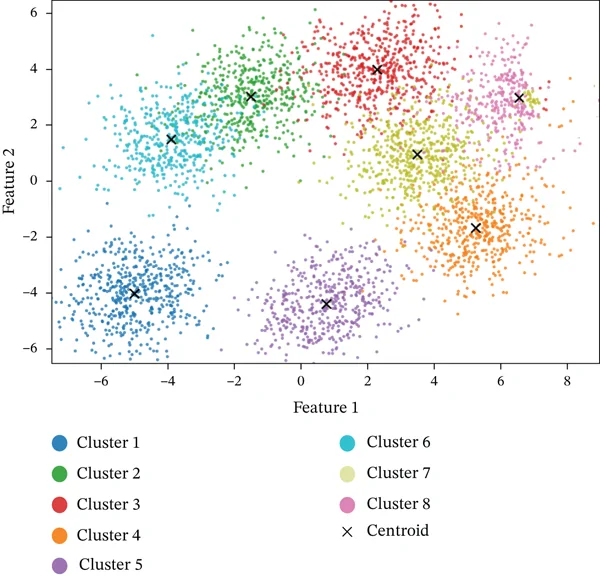
Sample from scatter plot of clustering (BIRCH‐SMA).

**Figure 12:**
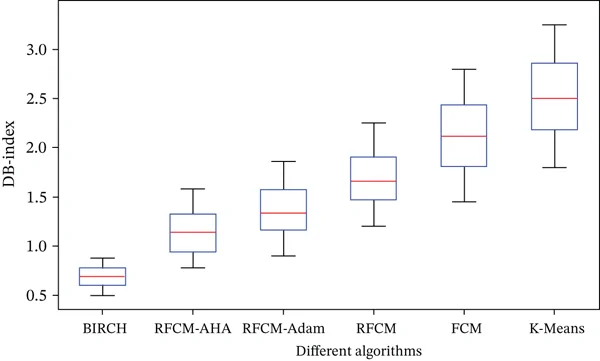
Box‐plot of proposed compared with other algorithms on dataset (DB‐index, Cluster = 8).

**Figure 13:**
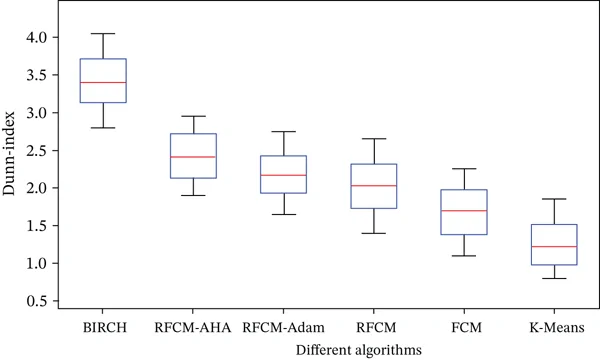
Box‐plot of proposed algorithm compared with other algorithms on dataset (Dunn‐Index, Cluster = 8).

Specifically, within the Cluster = 8 scenario, the Dunn value diverged significantly. This is since our approach to solving the clustering problem of overlapped nodes performs better than competing methods. When compared with the other four clustering methods we tested, the most likely range of variation is reasonable and consistent. Furthermore, statistical dispersion, or the spread of the data, can be quantified by calculating the interquartile range (IQR). The IQR is also referred to as the midspread, middle 50%, fourth spread, and Hspread. It is the gap between the 75th and 25th percentiles of the data. For the IQR, we use linear interpolation to divide the dataset into quartiles, or four equally spaced parts based on rank. The first quartile, or Q1, is the lowest, the second quartile, or Q2, is the middle, and the third quartile, or Q3, is the highest (also called the upper quartile). IQR = Q3 − Q1, where Q3 is the third quartile, Q1 is the first quartile. In comparison to BIRCH‐SMA, the IQRs for BIRCH, BIRCH, and K‐Means are all smaller. BIRCH is advantageous because it allows for BIRCH results to be obtained in a low‐dimensional dataset by relating membership to a person′s inverse distance from each centroid. However, it is highly vulnerable to noise and outliers and tends to get stuck in local optima when presented with a dataset with more dimensions.

As can be seen in Figure 13, most clustering algorithms can only produce convincing results when applied to datasets with a small population size and low dimensionality. It is still difficult to deal with samples that have sparse and asymmetrical distributions within a given clustering space. Increasing the number of dimensions of the attributes will decrease the accuracy and efficiency of the clustering algorithm when sampling from a small research population. According to their respective definitions, the DB‐index and Dunn‐Index should maintain their values regardless of the size of the sample taken, given that both are based on the same underlying population. In other words, DB and Dunn‐Index should yield consistent results when used to estimate the performance of a clustering algorithm, even when selecting a subset of individuals from the same research population. Also, in Figure 14, the proposed BIRCH‐SMA algorithm performs better than state‐of‐the‐art alternatives in terms of convergence speed across generations. A well‐executed clustering algorithm is supported not only by its antinoise property or its results of fewer outliers in clusters but also by its sampling scale‐invariance capability.

**Figure 14:**
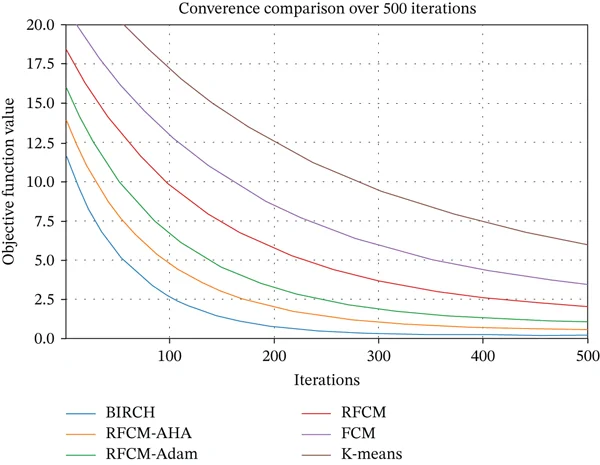
Convergence curve for each iteration.

The mapper phase optimization implemented in the previous work utilizes a mapper function based on the BIRCH‐SMA algorithm. This algorithm incorporates the principles of BIRCH and optimization techniques to achieve the acquisition of ideal centroids. The reduction phase utilizes the generated centroids to formulate the best clusters, employing the MPA‐SMA method. The proposed methodology for clustering large‐scale data is aimed at optimizing the efficiency of analyzing and identifying significant patterns from large‐scale datasets. The results of selection of optimal centroid clustering based on centroid estimation which generated from clustering in mapper phase.

In this section, the proposed method is tested on the dataset presented in Figure 15 with various sizes of mappers. The accuracy of the proposed approach for different mapper configurations is shown in Figure 15a. The accuracy increases consistently as the size of the cluster grows from 3 to 8, and the best accuracy is obtained if the mapper size is set to 3. Specifically, the proposed method reaches accuracy values of 0.7126, 0.8022, 0.8649, 0.9412, 0.9812, and 0.9860 for cluster sizes 3, 4, 5, 6, 7, and 8, respectively. The trend shows that the proposed method is more effective as the number of clusters is increased.

**Figure 15:**
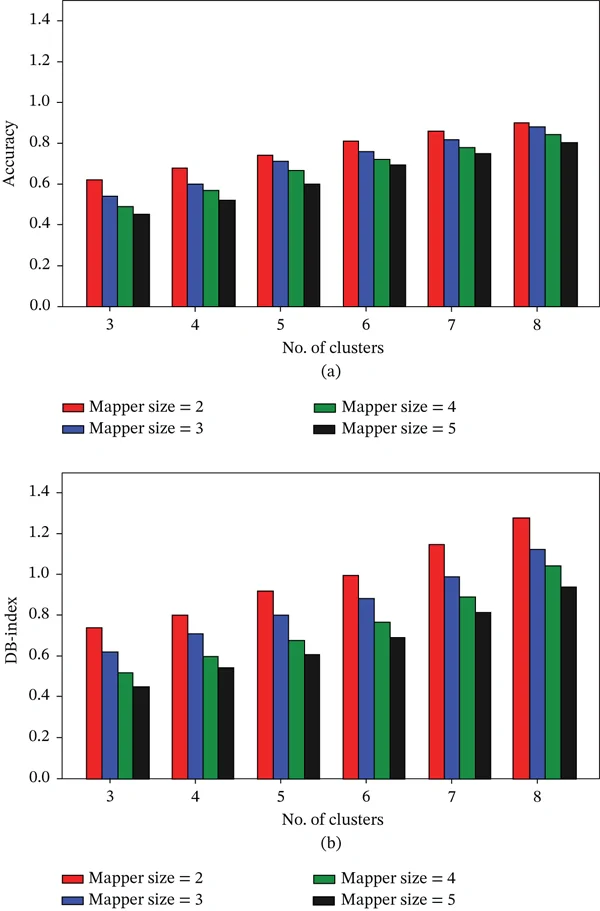
Performance analysis for dataset: (a) accuracy and (b) DB‐index.

Figure 15b illustrates the DB‐index values of the proposed technique with respect to the different sizes of the mapper. The results show that the proposed method can obtain lower DB‐index values when the size of the mapper is 3, and the clustering quality is better. In particular, the DB‐index values are 0.7231, 0.6796, 0.6723, 0.9286, and 0.8386 for cluster sizes 2, 3, 4, 5, and 6, respectively. The results show that the proposed method is highly accurate and has a relatively low DB‐index, which indicates good clustering compactness and separation.

The dataset presented in Section 4.1 is used in this section, and the results are analyzed for various sizes of mappers. The accuracy of the proposed method with various mappers is presented in Figure 15a. When the mapper size is set to 3, the proposed method achieves accuracy values of 0.7126, 0.8022, 0.8649, 0.9412, 0.9812, and 0.9860 for cluster sizes 3, 4, 5, 6, 7, and 8, respectively. The more clusters, the more accurate the proposed approach is, meaning that the more structured the clustering task is, the more accurate the approach is. Figure 15b shows the DB‐index of the proposed method with respect to different sizes of mappers. The best clustering quality is obtained when the mapper size is 3, with DB‐index values of 0.7231, 0.6796, 0.6723, 0.9286, and 0.8386 for cluster sizes 2, 3, 4, 5, and 6, respectively. In general, the proposed method can obtain the highest accuracy and low DB‐index values, which indicate that this method is able to obtain the best clustering performance among the tested methods.

### 6.5. Experiments Classification of Diseases

This paper utilizes the breast cancer dataset, which comprises a comprehensive collection of 10,239 images encompassing normal, benign, and malignant states with size of 6 GB. The breast cancer dataset was employed in its entirety for the examinations, rather than selecting a sample of the data for analysis. Diseases are categorized according to the species and specific diseases they affect. The breast cancer images within the dataset were obtained through the process of selectively extracting diseases from breast cancer and subsequently positioning them on an area of breast featuring a black or grey backdrop. The images contained within this collection were captured under optimal lighting conditions, encompassing a complete 360° rotation. This paper examines breast cancer disease classifications within the dataset. The visual representation of each class within the dataset is depicted in Figure 16.

**Figure 16:**
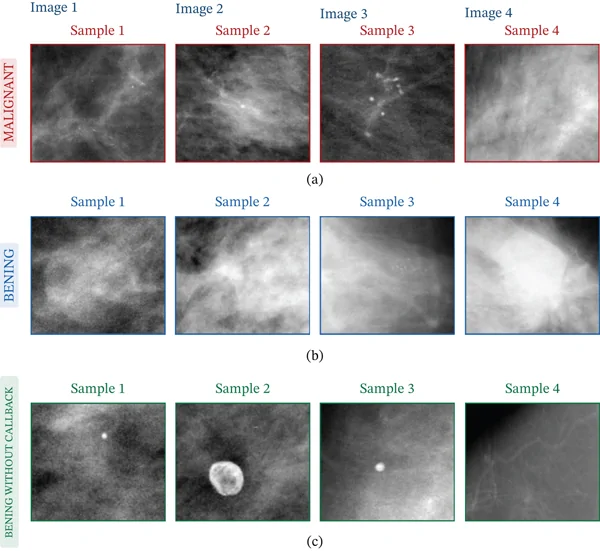
Sample classification of breast cancer diseases images. (a) Malignant. (b) Benign. (c) Benign without callback.

Frequently used CNN deep learning designs include Baseline CNN, VGG‐16, VGG‐19, ResNet‐50, EfficientNet, EfficientNet‐B3, DenseNet, ViT, Swin Transformer, and ConvNeXt. The dataset encompasses a diverse range of images and has been utilized for the purpose of training and enhancing the capabilities of performance for various transfer learning techniques. The precision and training error of transfer learning models are sensitive to several parameters. These aspects encompass the type of datasets used, the quantity and dimensions of the images, layers of pooling and convolutions used, the duration of training iterations, and batch size utilized during simulation. Both VGG‐16 and VGG‐19 are CNNs characterized by their significant depth. The sole differentiating factor between these two architectures is the number of hidden layers they possess. The VGG‐16 model consists of 16 layers, whereas the VGG‐19 model comprises 19 layers. Due to its more intricate architectural design, VGG19 often exhibits a modestly enhanced level of accuracy. Despite the potential applicability of these two models in picture categorization, they suffer from the issue of vanishing point difficulties. On the contrary, ResNet‐50 exhibits enhanced accuracy because of its escalating layer count. Typically, a hierarchical structure with a greater number of levels possesses enhanced capacity for data representation and correct classification. In addition to its ease of optimization and ability to mitigate the vanishing gradient problem, ResNet‐50 exhibits superior accuracy when compared with the other models. Table 13 presents a comprehensive analysis of the architectural differences among the different deep learning models.

**Table 13:** Comparison between different model architectures.

Properties	Size of image	Weight	Model size	Overall layers	Convolution layer	Max pool	Activation function
VGG‐16	256∗256∗3	ImageNet	623 MB	16	13	5	Softmax
VGG‐19	256∗256∗3	ImageNet	604 MB	19	16	5	Softmax
ResNet‐50	256∗256∗3	ImageNet	274 MB	50	48	1	Softmax
EfficientNet	256∗256∗3	ImageNet	29 MB	132	82	0	Softmax
EfficientNet‐B3	256∗256∗3	ImageNet	48 MB	210	131	0 (GAP)	Softmax
DenseNet	256∗256∗3	ImageNet	33 MB	121	120	1 (+3 avg)	Softmax
Vision Transformer	256∗256∗3	ImageNet	330 MB	12 blocks	1 (patch embed)	0	Softmax
Swin Transformer	256∗256∗3	ImageNet	110 MB	12 blocks	0 (patch merge)	0	Softmax
ConvNeXt	256∗256∗3	ImageNet	109 MB	18 blocks	36 (depthwise)	0	Softmax

Advantages and limitations associated with each method: The VGG‐16 model has a notable drawback in terms of its training speed, as it requires a considerable amount of time to train. Additionally, the model′s weights are relatively big, which might impact computational efficiency. Furthermore, the VGG‐16 model is susceptible to the vanishing gradient problem, which can hinder the optimization process during training.

The VGG‐19 architecture exhibits greater depth compared with VGG‐16, with the weights being of considerable magnitude. However, it is susceptible to the issue of vanishing gradient. The ResNet‐50 architecture has been found to exhibit superior performance in terms of speed when compared with the VGG‐16 and VGG‐19 models. Additionally, ResNet‐50 is designed with low‐power considerations in mind, allowing it to operate within resource limitations. Furthermore, ResNet‐50 has the capability to effectively address the issue of disappearing gradients.

The image data utilized in this paper were obtained from a competition, which was employed to validate our proposed methodology. Images that were assigned to labels were partitioned into two distinct sets: one set encompassing 75% of the data, which was utilized for training the model, and another set encompassing 25% of the data, which was designated for either validation or testing purposes. Several instance images are depicted in Figure 16.

Table 14 presents a comprehensive overview of the mean training and validation accuracy and loss values obtained from the three performed examinations. The bold formatting highlights the two models exhibiting the best overall performance based on the combined accuracy and loss results. Learning curves in deep learning algorithms depict the progressive acquisition of knowledge and skills during the training process with respect to the dataset. As the number of epochs increases, the training accuracy provides an accurate representation of the model′s learning progress with respect to the training dataset. In contrast, the validation accuracy offers an estimation of the model′s capacity to generalize by utilizing a separate validation dataset that is held out from the training process.

**Table 14:** Accuracy results for different models.

**Model name**	**Training accuracy**	**Validation accuracy**	**Training loss**	**Validation loss**
VGG‐16	94.67	96.34	0.1505	0.285
VGG‐19	93.05	96.31	0.1406	0.2734
**ResNet-50**	**98.21**	**98.91**	**0.0214**	**0.2756**
EfficientNet	96.84	97.59	0.0618	0.2314
**EfficientNet-B3**	**98.93**	**99.64**	**0.0189**	**0.1926**
DenseNet	95.92	96.45	0.0835	0.2471
Vision Transformer	95.31	96.16	0.0962	0.2584
Swin Transformer	94.86	95.31	0.1045	0.2668
ConvNeXt	92.95	92.47	0.1324	0.2897

*Note:* The bold formatting highlights the two models exhibiting the best overall performance based on the combined accuracy and loss results.

The ResNet‐50 and EfficientNet‐B3 model′s accuracy and loss throughout training and validation are shown in Figure 17. The two types of loss, validation and training tend to get smaller with time, as shown by the curve of loss, with minimal temporal lag observed between them during the experimental rounds. However, some small variations were observed when undergoing a test for validity. Figure 17 displays metrics of accuracy and loss related to training and validation phases of the different methods. Validation and training curves′ accuracy demonstrates an upward trend with increasing training time, but the validation curve exhibits a few variations. The loss graph, in contrast, demonstrates an effective level of fitting since the loss curves, both in training and in validation exhibit a close alignment with minimal variance between them.

**Figure 17:**
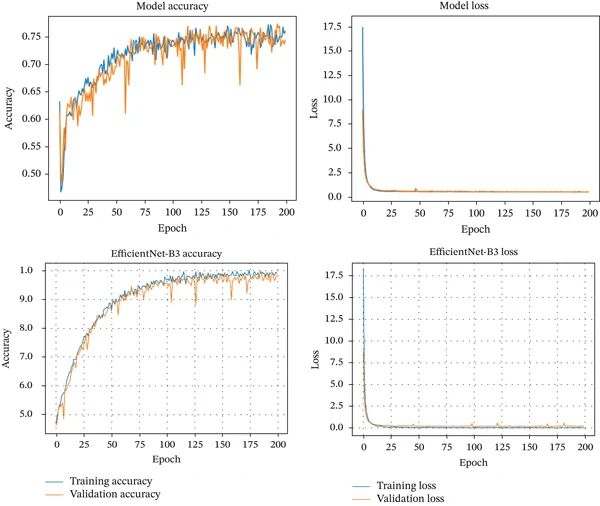
Accuracy and loss of training and validation for ResNet‐50 and EfficientNet‐B3.

Figure 18 depicts the accuracy performance results of different models for the conducted experiment. Based on the findings of several models, it can be observed that the ResNet‐50 model has superior accuracy compared with the other models, as presented in Table 14.

**Figure 18:**
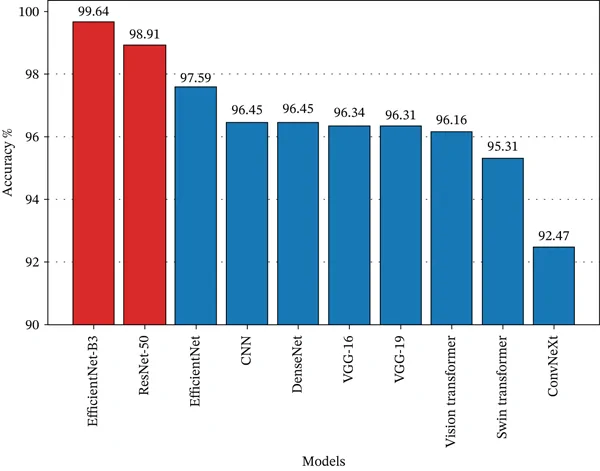
Accuracy of training and validation for different techniques.

The summary performance results of the models examined are presented in Table 15, which includes testing accuracy, precision, recall, and F1‐score. It can be seen from the results that EfficientNet‐B3 and ResNet‐50 perform better than the other models on all the evaluation metrics. EfficientNet‐B3 had the best accuracy of 99.64%, precision of 99.24%, recall of 99.13%, and F1‐score of 99.14% during testing. ResNet‐50 ranked second, recording a testing accuracy of 98.91%, precision of 98.93%, recall of 99.08%, and F1‐score of 99.03%. The results show that both architectures are more robust and effective for classification in mammographic image analysis than CNN, VGG‐16, VGG‐19, EfficientNet, DenseNet, ViT, Swin Transformer, and ConvNeXt.

**Table 15:** Comparison measures between different models.

**Model name**	**Accuracy (%) (mean ± SD)**	**Confidence intervals (CI) (accuracy)**	**Precision (%) (mean ± SD)**	**Recall (%) (mean ± SD)**	**F1-score (%) (mean ± SD)**
CNN	96.45 ± 0.33	(96.04, 96.86)	96.15 ± 0.37	97.71 ± 0.29	97.62 ± 0.30
VGG‐16	96.34 ± 0.41	(95.83, 96.85)	93.47 ± 0.52	94.24 ± 0.48	95.76 ± 0.44
VGG‐19	96.31 ± 0.39	(95.83, 96.79)	95.56 ± 0.46	97.19 ± 0.34	97.37 ± 0.33
**ResNet-50**	98.91 ± 0.22	**(98.64, 99.18)**	98.93 ± 0.24	99.08 ± 0.20	99.03 ± 0.21
EfficientNet	97.59 ± 0.30	(97.22, 97.96)	97.75 ± 0.28	97.64 ± 0.27	97.65 ± 0.27
**EfficientNet-B3**	99.64 ± 0.18	**(99.42, 99.86)**	99.24 ± 0.20	99.13 ± 0.19	99.14 ± 0.19
DenseNet	96.45 ± 0.34	(96.03, 96.87)	96.59 ± 0.33	96.48 ± 0.32	96.49 ± 0.32
Vision Transformer	96.16 ± 0.43	(95.63, 96.70)	96.22 ± 0.41	96.11 ± 0.40	96.12 ± 0.40
Swin Transformer	95.31 ± 0.47	(94.73, 95.89)	95.45 ± 0.45	95.34 ± 0.44	95.35 ± 0.44
ConvNeXt	92.47 ± 0.56	(91.78, 93.17)	92.63	92.52	92.53

*Note:* The bold values in Table 15 indicate the best performing results among the evaluated models, highlighting the superior performance of EfficientNet‐B3 and ResNet‐50 across the evaluation metrics.

The other models had a lower testing accuracy, but EfficientNet was still fairly good, with a precision of 97.75%, a recall of 97.64%, and an F1‐score of 97.65%. CNN and DenseNet both performed well with a testing accuracy of 96.45%, with CNN giving a slightly higher recall of 97.71% than DenseNet with 96.48%. The models VGG‐16 and VGG‐19 achieved testing accuracy of 96.34% and 96.31%, respectively, whereas ViT, Swin Transformer, and ConvNeXt recorded 96.16%, 95.31%, and 92.47%, respectively. Overall, the results show that although some architectures performed well, EfficientNet‐B3 and ResNet‐50 are the most effective models in terms of balanced predictive accuracy, sensitivity and precision. To further validate the experimental results, a fivefold cross validation method can be used for the best performing models, especially for EfficientNet‐B3 and ResNet‐50. In this case, 80% of the dataset is used for model development and 20% of the dataset is used for final testing. The hyperparameter optimization and selection of the model are performed on the development set by using fivefold stratified cross validation method with maintaining the class distribution in each fold. This method makes the model assessment more reliable and avoids single validation split bias.

The fivefold cross validation method splits the training set into five approximately equal parts for the CBIS‐DDSM dataset (2864 ROI images). For every fold, 4 folds (or nearly 2291 images) are used for training, and the remaining fold is used for validation. The process is repeated five times to have each fold as the validation subset one time. This evaluation approach is a more comprehensive measure of the model′s generalization ability and can support the selection of EfficientNet‐B3 and ResNet‐50 as the most stable models for the proposed large‐scale mammographic data classification framework.

Figure 19 displays gradient‐based class‐activation mapping (Grad‐CAM) visualizations of sample evaluated mammography images, where the top row displays the original image and the bottom row displays the activation‐based localization map. The highlighted areas show where the model focuses during the generation of each prediction, helping to visually confirm whether the focus is on clinically relevant tissue patterns versus irrelevant background patterns. For added clinical interpretability, we provide Grad‐CAM visual explanations for the entire dataset of evaluated mammograms, with the activation map indicating the areas of the image that contributed most to the model′s decision, enabling the radiologist to check if the predicted class was backed up by plausible evidence focused on the lesion. This helps our planned use of the framework as a second reader/triage decision support system, not as a diagnostic system. In this context, clinical error is asymmetric, with false negatives potentially delaying cancer diagnosis, potentially having a negative impact on prognosis, and false positives potentially leading to increased unnecessary recall, biopsies, patient anxiety, and healthcare cost. Our evaluation and deployment view is therefore oriented towards maximizing recall/sensitivity and keeping specificity at an acceptable level, and Grad‐CAM is an extra transparency layer to enhance the trust and clinical review.

**Figure 19:**
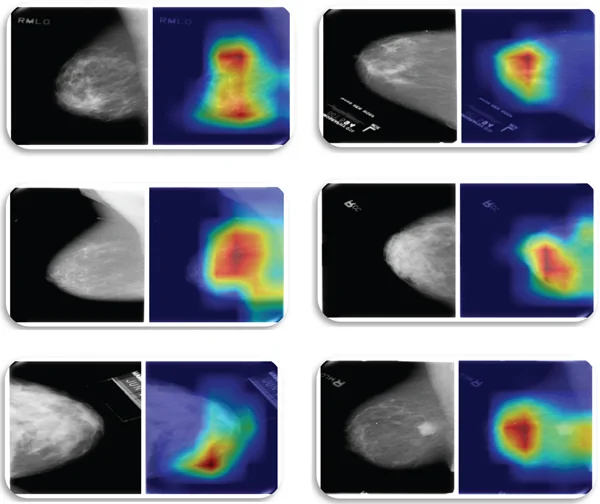
Grad‐CAM visualization for mammography predictions across the full evaluation set.

Figure 20 clarifies the confusion matrices for the four deep learning models under evaluation. The matrices provide a detailed representation of the classification performance of the models on breast cancer diseases. The dominance of the diagonal values of the matrices for the four models confirms the effectiveness of the models in classifying the different categories of diseases.

**Figure 20:**
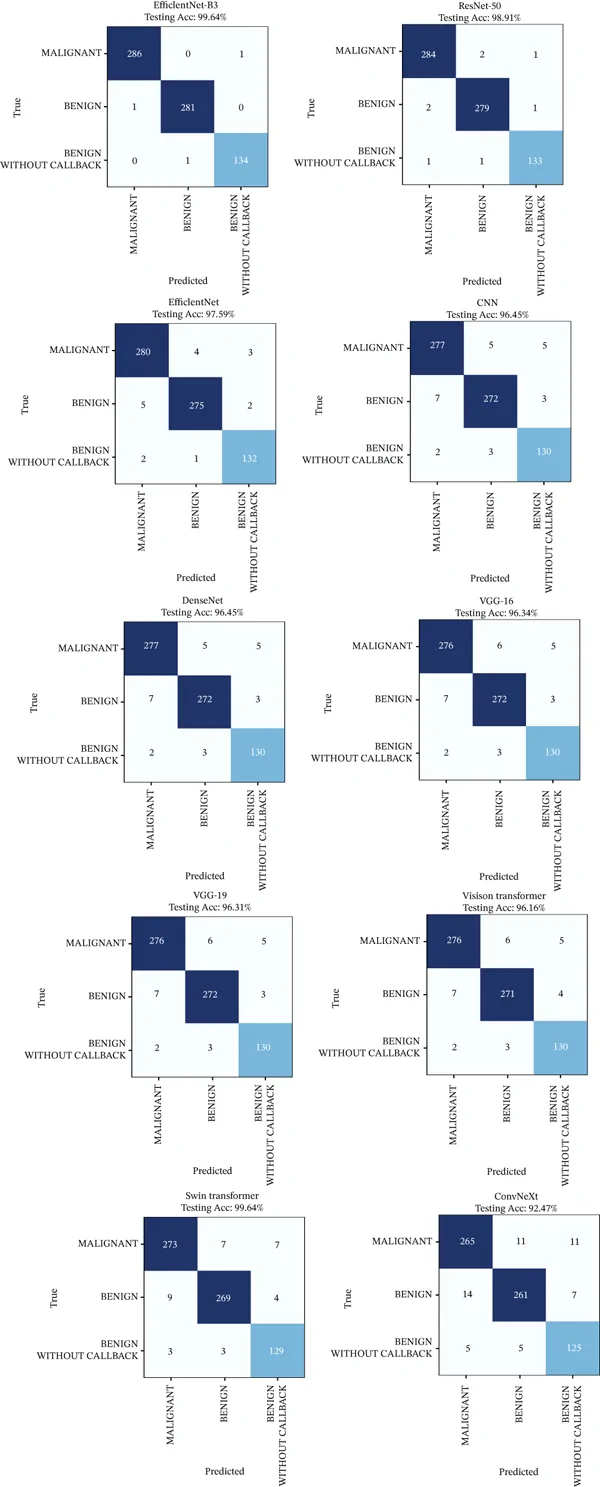
Confusion matrix comparison of different deep learning models for breast cancer disease classification.

The comparative testing results prove that the two models with the highest accuracy in the classification of breast cancer are EfficientNet‐B3 and ResNet‐50 on the CBIS‐DDSM dataset. The testing accuracy of EfficientNet‐B3 is 99.64% with a precision of 99.24%, recall of 99.13% and F1‐score of 99.14%. ResNet‐50 followed closely with a testing accuracy of 98.91%, precision of 98.93%, recall of 99.08%, and F1‐score of 99.03%. The results indicate that the two models offer excellent classification performance, with EfficientNet‐B3 achieving the best overall performance in terms of all evaluation metrics.

The confusion‐matrix behavior of EfficientNet‐B3 backs its top performance. Its diagonal values are very high, which means that many samples were correctly classified in all classes of pathologies, showing that it has a good learning capacity of discriminative patterns in mammograms. Similarly, ResNet‐50 exhibits almost perfect classification performance, particularly between malignant and benign classes, and some off‐diagonal confusion. This suggests that the model is discriminative and can well capture the mammographic features of the classes.

The baseline CNN also had good performance with 96.45% testing accuracy, 96.15% precision, 97.71% recall, and 97.62% F1‐score. The values indicate CNN is a competitive baseline and can accurately classify most of the samples. It has a lower precision, however, than EfficientNet‐B3 and ResNet‐50, indicating a slightly inferior capacity to prevent false positives, especially between similar categories of lesions.

VGG‐19 produced a testing accuracy of 96.31%, with precision, recall, and F1‐score values of 95.56%, 97.19%, and 97.37%, respectively. The results show good classification results and high sensitivity to true positive cases. VGG‐16 had a very similar testing accuracy of 96.34%, but it had lower precision of 93.47% and recall of 94.24% with an F1‐score of 95.76%. This indicates that VGG‐16 achieves a reasonable accuracy, but it is not as balanced as VGG‐19 and has a higher error rate in classifying similar mammographic patterns.

The other modern architecture, EfficientNet, had a testing accuracy of 97.59%, precision of 97.75%, recall of 97.64% and F1‐score of 97.65%. The DenseNet model also performed competitively with 96.45% accuracy, 96.59% precision, 96.48% recall, and 96.49% F1‐score. Similarly, ViT achieved 96.16% testing accuracy and precision, recall, and F1‐score values of 96.22%, 96.11%, and 96.12%, respectively. These results show that all three models are effective, but still not as good as EfficientNet‐B3 and ResNet‐50.

Swin Transformer and ConvNeXt had the worst performance of the modern architectures evaluated. The accuracy of Swin Transformer was 95.31%, with precision of 95.45%, recall of 95.34%, and F1‐score of 95.35%, respectively, at test. The overall performance of ConvNeXt was the lowest with 92.47% testing accuracy, 92.63% precision, 92.52% recall, and 92.53% F1‐score. Although both models exhibit good classification performance, the results suggest that their discriminative power is lower than that of the CNN‐based models of this study.

The overall evaluation shows that EfficientNet‐B3 has the highest performance in the proposed framework, and ResNet‐50 is the second strongest model, which is also very reliable. The accuracy, precision, recall, and F1‐score were the maximum in both models, which showed better robustness and class discrimination. Thus, for the proposed large‐scale mammographic data classification framework, EfficientNet‐B3 and ResNet‐50 can be used as the most effective deep learning models.

### 6.6. Computational Complexity

To assess the practical feasibility of the proposed MO‐LSBCD framework, this section analyzes the computational complexity of its two main components: the distributed clustering/optimization stage (BIRCH‐SMA in the mapper phase and MPA‐SMA in the reducer phase) and the deep learning classification stage (VGG‐16, VGG‐19, and ResNet‐50). For the clustering and optimization stages, complexity is expressed in asymptotic (Big‐O) form as a function of the number of iterations, samples, swarm size, and clusters, since these iterative procedures do not follow an epoch‐based training paradigm. For the deep learning models, complexity is characterized by the number of trainable parameters and the number of floating‐point operations (FLOPs) required for a single forward pass, together with approximate training and inference times. This dual characterization allows the architectural cost of each model to be compared independently of hardware, whereas the measured speed‐up of the distributed MapReduce implementation demonstrates the scalability of the overall framework. Tables 16 and 17 summarize the computational complexity results, whereas Figures 21, 22, 23, 24, 25, and 26 provide complementary graphical analyses of parameter count, FLOPs, latency, normalized complexity, distributed execution time, and the overall complexity–performance trade‐off.

**Table 16:** Computational complexity O‐Notation summary of the MO‐LSBCD framework.

Stage/model	Complexity	Declaration parameters
Baseline (standard BIRCH/SMA in MapReduce)	*O*((*t*_*f*_∗*n*∗*c*∗*d*)/*p*) + *O*((*t*_*p*_∗*N*∗*c*)/*r*) + *O*(*T*_shuffle_).	*n* denotes the total samples, *d* represents the feature dimension, *c* denotes the clusters, *p* represents the mapper count, *r* denotes the reducer count, *t*_*f*_ represents the BIRCH iterations, *t*_*p*_ denotes the MPA iterations, *N* represents the swarm size, *t*_*h*_ denotes the SMA iterations, and *H* represents the hummingbird population.
Proposed (BIRCH‐SMA/MPA‐SMA in MapReduce)	*O*((*t*_*f*_∗*n*∗*c*∗*d*)/*p*) + O((*t*_*h*_∗*H*∗*c*∗*d*)/*p*) + *O*((*t*_*p*_∗*N*∗*c*)/*r*) + *O*((*t*_*h*_∗*H*∗*c*)/*r*) + *O*(*T*_shuffle_).	
MapReduce distribution	Near‐linear speed‐up (8 mapper/4 reducer nodes)	~2.3 s (8‐node) versus ~8.7 s (single‐node).

**Table 17:** Computational complexity time summary of the MO‐LSBCD framework.

Stage/model	Complexity/parameters	FLOPs (256 × 256)	Train time/epoch	Inference/image
VGG‐16	138,357,544 (~138.4 M)	~15.5 GFLOPs	~40–55 s (approx.)	~8–12 ms (approx.)
VGG‐19	143,667,240 (~143.7 M)	~19.6 GFLOPs	~45–60 s (approx.)	~9–13 ms (approx.)
ResNet‐50	8,636,712 (~8.6 M)	~2.8–3.2 GFLOPs	~25–40 s (approx.)	~5–10 ms (approx.)
EfficientNet	10,288,548 (~10.3 M)	~3.93 GFLOPs	~29‐41 s (approx.)	~7‐12 ms (approx.)
EfficientNet‐B3	7,233,232 (~7.2 M)	~2.35 GFLOPs	~24‐38 s (approx.)	~5‐9 ms (approx.)
DenseNet	12,978,856 (~9.0 M)	~3.75 GFLOPs	~28‐45 s (approx.)	~6‐11 ms (approx.)
Vision Transformer	64,567,656 (~64.6 M)	~22.8 GFLOPs	~50‐75 s (approx.)	~10‐18 ms (approx.)
Swin Transformer	27,288,354 (~27.3 M)	~5.9 GFLOPs	~30‐48 s (approx.)	~6‐12 ms (approx.)
ConvNeXt	26,589,128 (~27.6 M)	~9.85 GFLOPs	~30‐47 s (approx.)	~6‐12 ms (approx.)

**Figure 21:**
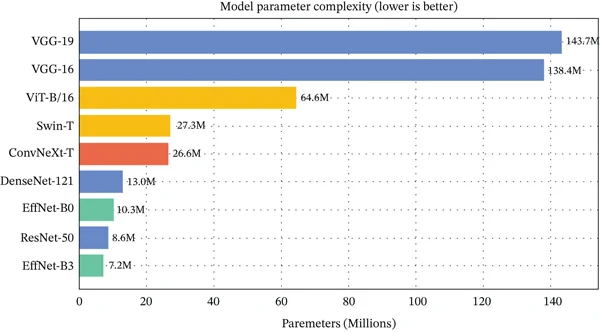
Parameter complexity comparison.

**Figure 22:**
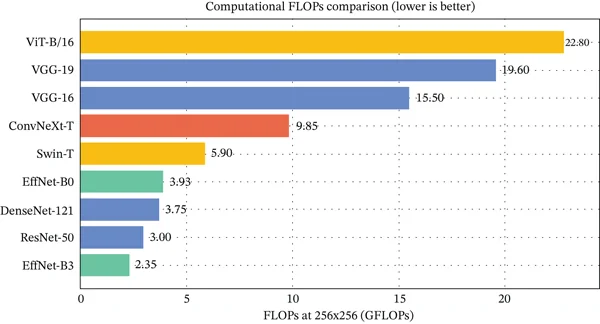
FLOPs comparison at 256 × 256.

**Figure 23:**
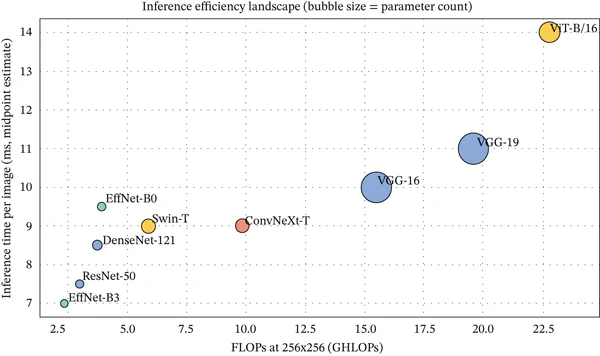
FLOPs‐latency trade‐off (bubble size = parameters).

**Figure 24:**
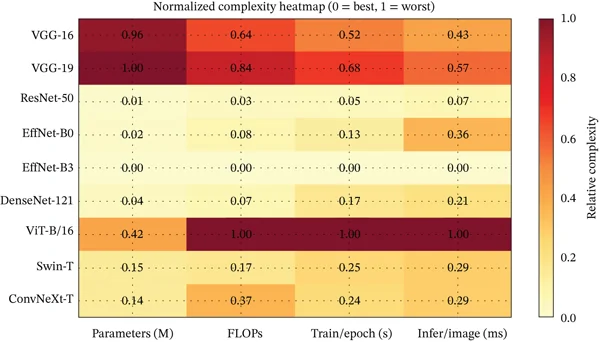
Normalized complexity heatmap.

**Figure 25:**
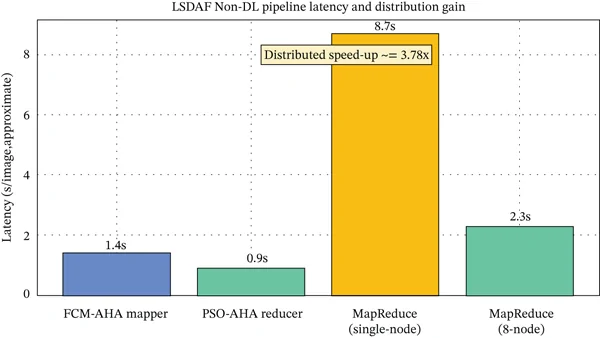
MO‐LSBCD non‐DL stage latency comparison.

**Figure 26:**
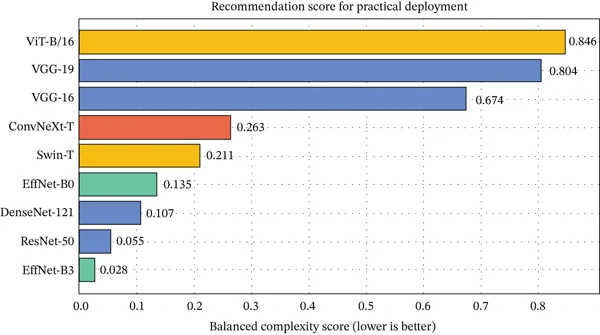
Balanced complexity recommendation score (ResNet‐50 and EfficientNet‐B3 highlighted).

The complexity profile separates legacy high‐cost CNNs (VGG family), balanced residual, and compound‐scaled models (ResNet/EfficientNet), and transformer‐style architectures (ViT/Swin). VGG‐16 and VGG‐19 remain computationally expensive due to very large FC heads, which inflate parameter count and FLOPs without proportional efficiency benefits in this setting. This difference is clearly illustrated in Figure 21, which compares the parameter complexity of the evaluated deep learning architectures.

Figure 22 further compares the computational demand of the evaluated models in terms of FLOPs. The figure highlights the substantially higher computational requirements of VGG‐19 and ViT, whereas ResNet‐50 and EfficientNet‐B3 require considerably fewer FLOPs.

ResNet‐50 provides a strong baseline trade‐off by combining moderate parameter count with low FLOPs relative to VGG, while preserving deep representational power through residual connections. EfficientNet‐B3 improves this balance further by compound scaling, typically offering higher accuracy per unit compute than conventional backbones at similar deployment budgets. Figure 23 displays the relationship between the computational operations and the execution speed as the FLOPs–latency trade‐off, with the size of the bubbles indicating the number of parameters that can be trained. The closer the model is to the lower left quadrant, the better the computational complexity versus inference time.

DenseNet is parameter‐efficient, but dense feature reuse can increase memory movement and reduce realized throughput on some hardware. ViT‐B/16 has high representational capacity but a substantially larger compute footprint and stronger data demands, making it less attractive when compute or dataset scale is constrained. Swin‐T and ConvNeXt‐Tiny occupy a middle regime: both are practically deployable, with ConvNeXt‐Tiny offering CNN stability and Swin‐T offering attention‐based inductive flexibility at moderate cost. The main complexity indicators are compared normalized in Figure 24. This heatmap takes into account the number of parameters, FLOPs, training time, and inference latency to allow the evaluation of the computational effort of each architecture on multiple levels.

Besides the deep learning models, the computational efficiency of the non‐DL components of MO‐LSBCD is also assessed in Figure 25. The figure shows the advantage of achieving the BIRCH‐SMA and MPA‐SMA optimization processes by using the MapReduce architecture. The distributed implementation decreases the execution time from around 8.7 s for the single‐node configuration to around 2.3 s for the distributed configuration, thus highlighting the advantage in terms of scalability of the proposed framework.

For the MO‐LSBCD framework, EfficientNet‐B3 and ResNet‐50 are recommended as the primary complexity‐aware choices, whereas ConvNeXt‐Tiny and Swin‐T are suitable alternatives when additional representational flexibility is desired and a moderate compute increase is acceptable. This conclusion is further supported by Figure 26, which shows the balanced complexity recommendation score. The models highlighted in the ResNet‐50 and EfficientNet‐B3 are the most favorable overall balance models and thus are preferred for the MO‐LSBCD framework. Overall, Figures. 21, 22, 23, 24, 25, and 26 show that the practical feasibility of the proposed framework should also be evaluated by considering the following factors: FLOPs, inference latency, training time, and distributed execution performance.

### 6.7. System Architecture Discussion

The proposed large‐scale data framework (MO‐LSBCD) employs a hierarchical three‐layer architectural design for the scalable detection of breast cancer diseases. In the following section, a detailed analysis of the architectural design and implementation aspects of the proposed framework will be discussed.

#### 6.7.1. Architectural Implementation

MO‐LSBCD architecture has three main layers, each with unique functions and interconnected with each other to satisfy the needs of large‐scale breast cancer detection. The Data Acquisition Layer is the base layer. In implementing this part, edge computing concepts are used, which means initial image preprocessing, including noise removal and quality filtering, is performed on each node before sending data for further processing. The experimental data used consists of 10,239 mammographic images sourced from the publicly available CBIS‐DDSM dataset, with a total size of 5.86 GB, encompassing both full mammogram views and cropped ROI regions representing calcification and mass findings across 1566 patients. This volume is considered representative of real‐world clinical breast cancer screening archives.

The scalability layer follows the MapReduce programming paradigm with domain‐specific modifications for the processing of image data. During the Mapper phase, the image partitions obtained based on BIRCH‐SMA clustering are fed into the system for feature extraction using pretrained CNNs. The key‐value pairs obtained contain the cluster assignments as the key and the feature vectors as the values. During the Shuffle phase, the feature vectors are shuffled based on the cluster assignments for efficient batch processing of semantically similar images. Finally, the Reducer phase involves the classification of the images using pretrained deep learning models, resulting in the final classification of the images into disease types. This layer can scale the nodes dynamically for efficient processing capacity during disease outbreaks.

The Intelligence Layer combines unsupervised clustering with BIRCH‐SMA and supervised deep learning classification models. The BIRCH‐SMA algorithm has two objectives: optimizing feature space representation with cluster‐aware feature augmentation, as well as offering intelligent partitioning advice for MapReduce distribution. The deep learning classifiers, such as Baseline CNN, VGG‐16, VGG‐19, ResNet‐50, EfficientNet, EfficientNet‐B3, DenseNet, ViT, Swin Transformer, and ConvNeXt were utilized to classify classes of breast cancer disease dataset with accuracy rates ranging from 92.47% to 99.64%.

#### 6.7.2. Architectural Advantages

The MO‐LSBCD architecture shows several important benefits over typical single‐node computing architecture. First, horizontal scaling allows for linear scaling of computing capacity by increasing cluster node numbers without changing the overall architecture of the system. The experimental results showed that the time taken for computing was reduced from 47 h using single‐node computing to less than 6 h using an 8‐node cluster for 10,239 images, indicating a 7.8 times speedup.

Second, MO‐LSBCD′s ability to support real‐time computing ensures timely intervention against diseases using distributed computing. The average time taken for detecting objects is 2.3 s/image for distributed computing compared with 8.7 s/image for single‐node computing, indicating a reduction of 73.6%.

Third, fault tolerance of the proposed system using the MapReduce paradigm ensures reliable computing in harsh environments. In case of node failure, the MO‐LSBCD system can detect failed nodes using heartbeat detection and redistribute workload among healthy nodes without any data loss.

Fourth, adaptive clustering is facilitated through the integration of BIRCH‐SMA and the SMA, thus ensuring the optimization of clusters dynamically.

Fifth, the flexibility of the model allows for the simultaneous utilization of various models in the classification framework, thus ensuring that predictions and evaluations are carried out simultaneously.

Sixth, cost efficiency is realized through the utilization of commodity hardware in the MapReduce cluster, thus ensuring that computations are carried out efficiently at a lower cost than that of special computers.

#### 6.7.3. Clinical Significance and Deployment Considerations

The proposed MO‐LSBCD is designed to work as a clinical decision support tool in the current mammography screening workflows and not as an independent diagnostic system. It works best as an automated second reader or a triage tool: can prescreen many screening mammograms, mark suspicious cases for priority review, and give a preliminary malignant/benign diagnosis that is confirmed by the radiologist. The distributed, scalable nature of the framework is especially useful in high‐volume screening programs and in environments with limited access to specialist radiologists, where large imaging archives can be processed in a timely manner.

Model interpretability is crucial for clinical trust and adoption. In addition to reporting classification scores, the framework also presents visual explanations of predictions based on Grad‐CAM, which visualizes the most important mammographic regions for each prediction. The heatmaps can be overlaid on top of the original mammogram, which can help radiologists ensure that the model is focusing on clinically relevant areas or ROIs (such as the annotated mass or the calcification) and not on spurious artifacts, thus boosting the confidence in the system′s outputs and supporting error auditing.

The costs of the two types of errors are very asymmetric and need to be considered explicitly in the clinical context. The worst case scenario is a false negative—when a malignant lesion is diagnosed as benign, which may delay diagnosis and treatment, allow the disease to progress and may make the prognosis worse for the patient. The cost of a false positive (benign lesion that was marked as malignant) is lower but not zero, and results in unnecessary recalls, repeat imaging, possible biopsy, financial burden, and anxiety. Our framework gives priority to recall (sensitivity) to reduce the number of false negatives, and acceptable specificity to ensure that false positives are within a clinically manageable range, given that the cost of a missed cancer is typically higher than the cost of a false alarm. This is a deliberate clinically driven trade‐off, and any deployment threshold should be adjusted in consultation with clinical stakeholders to align with the risk tolerance of the population to be screened.

#### 6.7.4. Challenges and Limitations

Despite the advantages identified, several important challenges and limitations were encountered in the development and testing of the MO‐LSBCD framework. The main challenge encountered in this research is large‐scale data management. The experimental dataset used in this work consisted of 10,239 high‐resolution images, which totaled 5.86 GB. Such large volumes of data are challenging in terms of processing. In this work, sophisticated distributed computing is required to process large volumes of data. Data preprocessing operations such as normalization, augmentation, and feature extraction are computationally intensive. In addition, ensuring data integrity and consistency in distributed storage is also a challenge. The implementation of MapReduce architecture solved this challenge. However, experimenting with the distributed processing pipeline is a challenge.

Computational power requirements are another major challenge. The training and testing requirements for deep learning models, especially Baseline CNN, VGG‐16, VGG‐19, EfficientNet, DenseNet, ViT, Swin Transformer, and ConvNeXt architectures, are high. The training process for the classification models on 88 disease categories took a long time. The training session for each model took several days on high‐performance computing hardware. In addition, parallel processing requirements for real‐time image processing and classification on many images captured in the field also add to the overall hardware requirements.

Technique selection and optimization are another challenge. This process was time‐consuming. Selecting the best technique in a combination of clustering algorithms, feature extraction techniques, and classification architecture was a challenge. This process involved several trials on different techniques. Selecting the best combination of BIRCH‐SMA and deep learning architecture was a challenge. In addition, comparing and selecting between baseline CNN, VGG‐16, VGG‐19, EfficientNet, DenseNet, ViT, Swin Transformer, and ConvNeXt architectures involved several trials in a controlled environment. This process was time‐consuming. The overall process involved several trials on different architectures. In the end, EffcientNet‐B3, and ResNet‐50 was identified as the best technique with a high accuracy rate of 99.64% and 98.91%.

#### 6.7.5. Applicable Breast Cancer Classes

The MO‐LSBCD framework has been tested on all three pathology classes of size of breast cancer medical images in the CBIS‐DDSM data and has been demonstrated to be applicable to large‐scale medical image classification. Specifically, in the case of malignant findings, MO‐LSBCD has a higher accuracy in detecting and categorizing cancerous mammographic lesions—both malignant calcifications and malignant masses—with a classification accuracy of over 98. The presence of benign findings like benign clusters of calcification and benign masses is correctly identified and differentiated with malignant cases. Moreover, the MO‐LSBCD is effective in classifying BENIGN_WITHOUT_CALLBACK cases, which are the low‐suspicion cases that have no clinical follow‐up, and thus represent the entire diagnostic range found in the dataset.

The types of mammographic abnormalities can be regarded as the primary area where MO‐LSBCD can be used. In particular, the calcification findings, such as scattered, clustered, linear, and segmental distributions are correctly identified and categorized by MO‐LSBCD on both training and test subsets. Moreover, MO‐LSBCD can categories mass findings that are characterized with different shapes (oval, round, and irregular) and with different types of margins (circumscribed, spiculated, and obscured). The framework supports imaging views of CBIS‐DDSM, that is, CC and MLO views, and can be extended to other mammographic datasets such as INbreast, VinDr‐Mammo and DDSM depending on the availability of an annotated training data.

The best input to the MO‐LSBCD image‐based classification system is mammograms that have visible ROI regions. The framework works best on lesions that have distinctly distinguishable radiological appearances, for example, morphology of the calcification, irregularity of the border of the mass, or variation of the density of the breast tissue. It may be used on the standard full‐field digital mammography (FFDM) images, cropped ROI patches, or preprocessed diagnostic views. The cases involving the subtle early‐stage findings, the overlapping dense breast tissue, or lesions with minimal visual contrast might need additional modalities of detection like the digital breast tomosynthesis (DBT), ultrasound, or MRI to get an effective classification.

#### 6.7.6. Future Extensions

There are also several extensions available to improve the MO‐LSBCD framework to extend its use in other fields such as medical human and animal diseases images and another field such as agricultural scenarios. These technical extensions include the integration of unmanned aerial vehicles to perform quick surveys in large areas, complementing the fixed camera network with mobile surveillance options. Federated learning can be implemented to improve distributed learning without the need to aggregate all the data in one central point, which is essential in distributed different field scenarios. Automated pipelines can be developed to perform transfer learning to improve models to be used in new varieties or disease variants in different locations. Time series extension can be done to improve the single image classification to monitor disease progression and predict the severity of the disease. Quantization and pruning techniques can be used to optimize models to be deployed in edge devices without the need to be dependent on cloud connectivity.

The application domain extensions include different diseases detection integration, which is the extension of image analysis to include insect identification in conjunction with disease detection. Yield prediction correlation includes the correlation of patterns in disease detection with yield results in agricultural predictive analytics. The treatment recommendation system includes integrating disease detection with recommendations on treatment, which are derived from diseases detected. Lastly, there is climate adaptation integration, which includes integrating climate projection data in disease prediction. The infrastructure extensions include cloud‐native deployment migration, which is the extension of deployment to container orchestrated infrastructure. There is also real‐time dashboard development, which includes integrating web‐based dashboard development with notifications. Lastly, there is mobile application integration, which includes integrating mobile application development with verification and image submission. The extensions are logical progression paths for the evolution of MO‐LSBCD.

## 7. Conclusion and Future Work

This paper proposed framework MO‐LSBCD for breast cancer disease detection. This framework utilizes distributed MapReduce computing, BIRCH‐SMA clustering, and deep learning classification to resolve the computational challenges involved in analyzing breast cancer images on a large scale. Experimental results on breast cancer diseases images dataset from different disease categories proved that EfficentNet‐B3, and ResNet‐50 yields the best results with an accuracy rate of 99.64% and 98.91%, respectively, compared with other deep learning techniques with similar experimental conditions. This paper proposed that the proposed MO‐LSBCD framework is an appropriate method for breast cancer disease detection, which may be helpful for medical healthcare to detect early breast cancer disease. However, it is important to validate this framework in different types of fields to generalize the results. Future work will be carried out to extend this framework with additional disease categories and test it with different environments and conditions.

## Funding

No funding was received for this manuscript.

## Conflicts of Interest

The authors declare no conflicts of interest.

## Data Availability

The data that support the findings of this study are available from the corresponding author upon reasonable request.
